# Significance of Mutation Spots and Concurrent Gene Mutations on Prognosis and Clinical Outcomes in Myelodysplastic Syndromes With *SF3B1* Mutation

**DOI:** 10.1002/cam4.70930

**Published:** 2025-05-08

**Authors:** Qi Liu, Fanhuan Xu, Juan Guo, Feng Xu, Xinhui Huang, Jianan Chen, Jiacheng Jin, Liyu Zhou, Qi He, Dong Wu, Luxi Song, Zheng Zhang, Cha Guo, Jiying Su, Yumei Zhang, Meng Yan, Chunkang Chang, Xiao Li, Lingyun Wu

**Affiliations:** ^1^ Department of Hematology Shanghai Sixth People's Hospital Affiliated to Shanghai Jiao Tong University School of Medicine Shanghai China; ^2^ Department of Hematology Shanghai Eighth People's Hospital Shanghai China

**Keywords:** immunity, myelodysplastic syndromes, prognosis, *SF3B1* gene

## Abstract

**Purpose:**

To investigate the clinical characteristics and prognosis of mutation spots and concomitant gene mutations in myelodysplastic syndromes (MDS) with *SF3B1* mutation (*SF3B1* ^*mut*^).

**Patients and Methods:**

Patients diagnosed with MDS at Shanghai Jiao Tong University School of Medicine Affiliated Sixth People's Hospital from October 2008 to November 2023 were enrolled in this study. *SF3B1* ^*mut*^ was identified by next‐generation sequencing (NGS).

**Results:**

One hundred and seven (8.7%) cases harbored the *SF3B1* mutation. The most frequent *SF3B1* ^*mut*^, noted in 47.66% of all patients, was the hotspot *K700E*. *K666* and *R625* were observed in 24.30% and 9.35%, respectively. Two less frequent mutation subtypes accounted for 5.61% of *H662* and 4.67% of *E622*. Patients with the *K666* mutation showed more severe thrombocytopenia (*p* = 0.032), significantly lower NK cell percentage (*p* = 0.001), and the Th1/Th2 ratio (*p* = 0.018) in the bone marrow (BM). The overall survival (OS) in patients with *E622* and *H662* mutations was significantly longer than that of patients with the *R625* mutation (*p* = 0.045) and the *K666* mutation (*p* = 0.010). Multi‐variance analysis showed the *SF3B1* mutation involving the *K666* hotspot independently predicted overall survival in MDS (HR 2.094, *p* = 0.050). Notably, most (11/13, 84.6%) of concomitant *TP53* mutations were mono‐hit, which did not affect the survival of patients in our cohort.

**Conclusions:**

*SF3B1* ^*mut*^ patients with specific mutation spots and concomitant gene mutations showed distinct clinical features and prognosis. Consequently, a comprehensive study of specific subtypes is of great significance for improving the prognosis of patients with *SF3B1* mutations.

## 1 Introduction

Myelodysplastic syndromes (MDS) are a group of clonal malignant diseases that originate from hematopoietic stem cells, which are characterized by ineffective hematopoiesis and cytopenia, with an increased risk of transforming into acute myeloid leukemia (AML) [1]. Genetic mutations are closely related to the pathogenesis of MDS, the most common of which include genes related to RNA splicing [2]. *SF3B1* is one of the most frequently mutated splicing genes in MDS, mutations of which may cause the abnormal gene splicing of hemoglobin synthesis and iron metabolism, and then result in the dysynthesis of hemoglobin and the formation of ringed sideroblasts (RS) [3, 4, 5]. The most common clinical characteristic is anemia. Multiple researches have shown that the *SF3B1* mutation (*SF3B1* ^*mut*^) is associated with a favorable prognosis and a lower possibility of transforming to AML. The International Working Group for the Prognosis of MDS has proposed *SF3B1* ^*mut*^ MDS as a distinct disease subtype [1]. In the 5th edition of the classification of hematolymphoid tumors (WHO‐HAEM5), MDS with low blasts and *SF3B1* mutation was defined as a new subtype [6].

*K700E* is the most frequent mutation spot in *SF3B1* ^*mut*^ MDS. Previous research has discovered that patients with the *K700E* mutation show a favorable prognosis, while those with the *K666* mutation are associated with a negative prognosis. However, information about the clinical features and prognosis of *non‐K700E* and *non‐K666* mutations in *SF3B1* mutant MDS is scarce. *SF3B1* ^*mut*^ MDS patients with complex mutational status (at least two associated mutations) are associated with a poor prognosis. Specific genes are associated with the unfavorable prognosis in MDS. More than 40% of patients with MDS have at least two mutations, and the co‐occurrence of particular genes may change the effect on the prognosis of a single mutation. IPSS‐M includes somatic mutations in 31 genes to provide a risk score. Previous studies have divided *SF3B1* ^*mut*^ MDS into three independent groups: *SF3B1* ^*5q*^, *SF3B1* ^*α*^, and *SF3B1* ^*β*^. The favorable outcomes are only confined to the *SF3B1* ^*α*^ group without mutations in *BCOR*, *BCORL1*, *RUNX1*, *NRAS*, *STAG2*, and *SRSF2*, as well as del (5q) [7]. However, little is known about the impact of *TP53* concurrent mutations on the prognosis of *SF3B1* ^*mut*^ MDS. In addition, the pathogenesis of MDS involves various factors, and it is not yet fully elucidated. With the exception of molecular genetics and cytogenetics, immunity has been confirmed to play a role in the pathogenesis of MDS. According to previous findings, the activation of inflammatory immune signaling is seen in *SF3B1* ^*mut*^ MDS. However, the immune characteristics of mutational subtypes in *SF3B1* ^*mut*^ MDS have not been explored. In the current study, the clinical features, immune cells in bone marrow (BM), and prognosis of patients with different *SF3B1* mutation spots will be explored. Furthermore, concomitant gene mutations, including the *TP53* mutation, will also be analyzed in our cohort for their prognostic value in *SF3B1* ^*mut*^ patients.

## 2 Materials and Methods

### 2.1 Patients

Patients diagnosed with MDS according to WHO 2016 [8] at Shanghai Jiao Tong University School of Medicine Affiliated Sixth People's Hospital from October 2008 to November 2023 who harbored the *SF3B1* mutation identified by next‐generation sequencing (NGS) were enrolled in this study. Clinical characteristics including age, sex, hemoglobin, platelet, absolute neutrophil count in the peripheral blood, blasts and RS in bone marrow (BM), cytogenetics, serum ferritin, and serum erythropoietin level were analyzed in this study. Follow‐up time started from the date of the diagnosis of MDS and ended on June 1, 2024. Overall survival (OS) was defined as the date of diagnosis to the date of death, end of follow‐up, or loss to follow‐up. Leukemia‐free survival (LFS) was defined as the time from disease diagnosis to progression to leukemia or death. The study was approved by the Ethics Committee of Shanghai Sixth People's Hospital Affiliated to Shanghai Jiao Tong University School of Medicine and was in accordance with the 1964 Helsinki Declaration and its later amendments or comparable ethical standards. Informed consent from all patients was obtained.

### 2.2 Targeted Gene Sequencing

Thirty‐eight genes, including *ASXL1, ANKRD11, BCOR, CALR, CBL, CEBPA, DNMT3A, ETV6, EZH2, FLT3, GATA2, IDH1, IDH2, ITIH3, JAK2, KIF20B, KIT, KRAS, MPL, NF1, NPM1, NRAS, PHF6, PTPN11, PTPRD, ROBO1, ROBO2, RUNX1, SETBP1, SF3B1, SRSF2, STAG2, TET2, TP53, U2AF1, UPF3A, WT1*, and *ZRSR*2 were examined for mutations by MiSeq sequencing (Illumina, San Diego, CA, USA) in gDNA from BM mononuclear cells of patients. Detailed operating steps were described in our previous studies [9]. [Correction added on July 28, 2025 after first online publication. The term ‘cDNA’ has been replaced with ‘gDNA’ in the previous sentence.]

### 2.3 Flow Cytometry Analysis

T cell subsets and NK cells in BM were detected with Coulter Epics‐XL (Beckman Coulter) and analyzed by System II Software (Beckman Coulter). The following antibodies were used in this study: PC5‐labeled anti‐CD3, ECD‐labeled anti‐CD8, fluorescein isothiocyanate (FITC)‐labeled anti‐human IFN‐γ, and phycoerythrin (PE)‐labeled anti‐human IL‐4 (Beckman Coulter). CD3^+^/CD8^−^ cells were defined as CD4^+^ cells. Th and Tc subsets were defined as Th1 (CD8^−^/INF‐γ^+^), Th2 (CD8^−^/IL‐4^+^), Tc1 (CD8^+^/INF‐γ^+^), and Tc2 (CD8^+^/IL‐4^+^). Detailed operating steps were described in our previous studies [10].

### 2.4 Enzyme‐Linked Immunosorbent Assay (ELISA)

The levels of serum erythropoietin and serum ferritin were measured by ELISA. The normal reference value of the level of serum erythropoietin is from 4.3 mIU/mL to 29.0 mIU/mL, and the normal reference value of the level of serum ferritin is from 30.0 ng/mL to 400.0 ng/mL.

### 2.5 Statistical Analysis

SPSS version 27.0 and GraphPad Prism 10.0 were used for statistical analysis. Continuous variables that followed the normal distribution were represented by the mean, and continuous variables that did not conform to the normal distribution were represented by the quartile. The differences between continuous variables were compared by the independent‐samples *t* test, one‐way analysis of variance, Mann–Whitney *U* test, and Kruskal–Wallis test. A chi‐square test was used to compare the differences between the categorical variables. The Kaplan–Meier curve was used for survival analysis. Log‐rank was used to test the difference in OS. Based on univariate analysis, variables with statistically significant differences were analyzed by Cox multivariate analysis. *p* < 0.05 was considered statistically significant.

## 3 Results

### 3.1 Clinical Characteristics of Mutation Spots in *SF3B1* ^*mut*^ MDS

A total of 1226 MDS patients were enrolled in this study, and 107 (8.7%) cases harbored *SF3B1* mutations. The median variant allele frequency (VAF) of the *SF3B1* gene was 39.2% (range: 2.5%–59.0%). There were 65 males and 42 females, with a median age of 66 years (range: 20–89 years). According to the 2016 WHO categories, 4.9% of patients were diagnosed as MDS with single lineage dysplasia (MDS‐SLD), 12.6% of patients were diagnosed as MDS with multilineage dysplasia (MDS‐MLD), 57.3% of patients were diagnosed as MDS with ring sideroblasts (MDS‐RS), 14.6% of patients were diagnosed as MDS with excess blasts‐1 (MDS‐EB‐1), 8.7% of patients were diagnosed as MDS with excess blasts‐2 (MDS‐EB‐2), 1.0% of patients were diagnosed as MDS with isolated deletions of the long arm of chromosome 5 (del [5q]), and 1.0% of patients were diagnosed as unclassifiable MDS (MDS‐U). Cytogenetic analysis showed that 43.9% of cases had the normal karyotype, 4.7% of cases had del (5q), 7.5% of cases had deletions of the long arm of chromosome 20 (del [20q]), 6.5% of cases had trisomy 8, 6.5% of cases had complex karyotypes, 2.8% of cases had abnormalities of chromosome 3, and 0.9% of cases had loss of chromosome 7. According to the revised International Prognostic Scoring System (IPSS‐R), patients were classified as very low‐risk group (4.3%), low‐risk group (33.7%), intermediate‐risk group (40.2%), high‐risk group (9.8%), and very‐high risk group (12.0%). Among IPSS‐R categories, 78.2% of cases were relatively low‐risk groups, including very‐low risk, low‐risk, and intermediate‐risk group, whereas 21.8% of cases were relatively high‐risk groups, including high‐risk and very‐high risk group. Among all patients, only 17.5% of patients progressed to AML.

**FIGURE 1:**
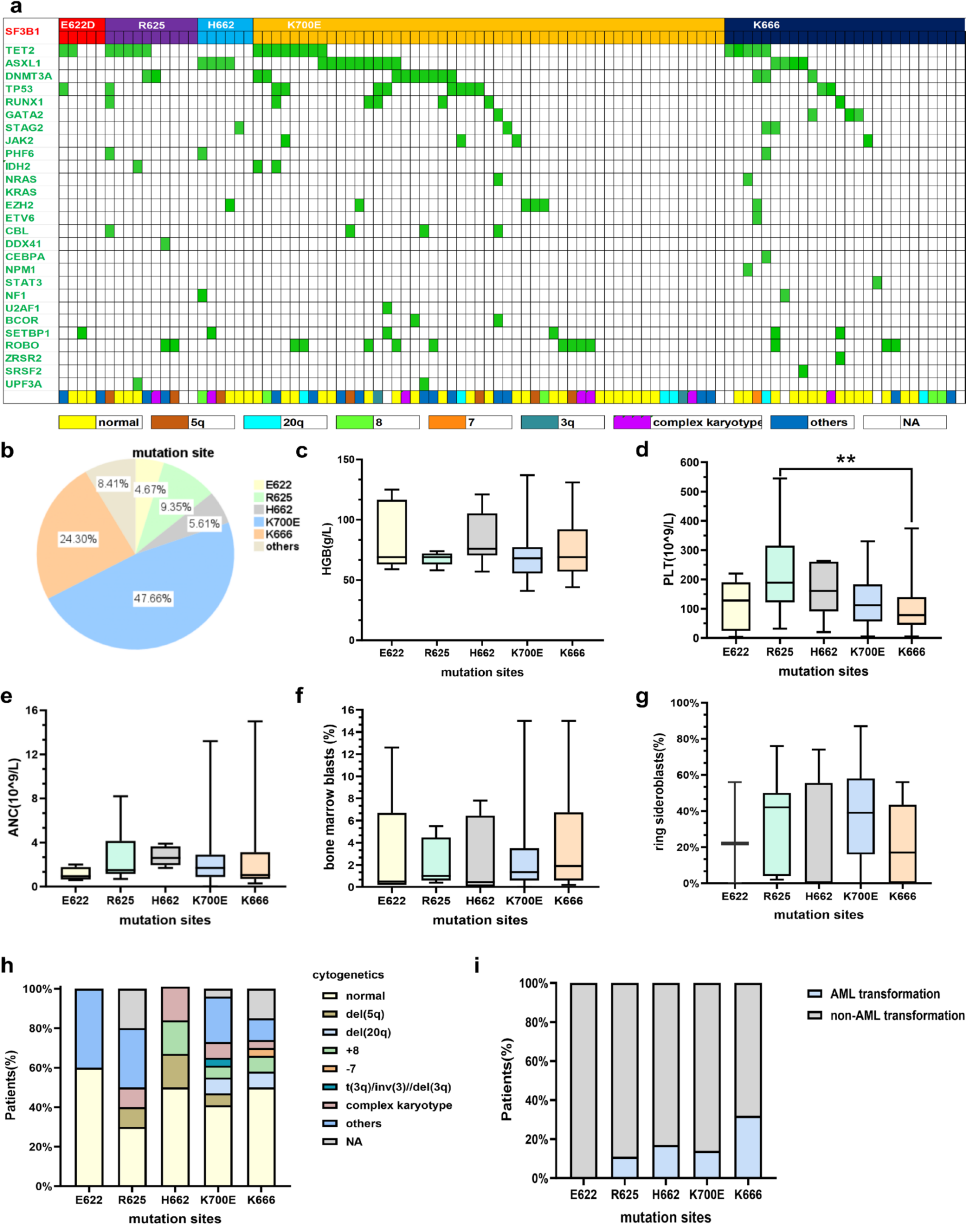
Mutation landscape and clinical characteristics of mutation spots in *SF3B1* ^*mut*^ MDS. (a) Mutation landscape of mutation spots in MDS patients with the *SF3B1* mutation. (b) The proportion of distinct mutation spots in MDS patients with the *SF3B1* mutation. (c) The difference in the level of hemoglobin in *SF3B1* ^*mut*^ MDS patients with distinct mutation spots. (d) The difference in the level of platelets in *SF3B1* ^*mut*^ MDS patients with distinct mutation spots. (e) The difference in the absolute neutrophil count in *SF3B1* ^*mut*^ MDS patients with distinct mutation spots. (f) The difference in the percentage of BM blasts in *SF3B1* ^*mut*^ MDS patients with distinct mutation spots. (g) The difference in the percentage of RS in *SF3B1* ^*mut*^ MDS patients with distinct mutation spots. (h) The cytogenetics in *SF3B1* ^*mut*^ MDS patients with distinct mutation spots. (i) The proportion of AML transformation and non‐AML transformation in *SF3B1* ^*mut*^ MDS patients with distinct mutation spots. **p = 0.010.

Further, we compared the clinical features of different mutation spots in *SF3B1* ^*mut*^ patients. Fifty‐one of 107 cases (47.66%) had the *K700E* mutation with the median VAF of 39.65%, 26 cases (24.30%) had the *K666* mutation with the median VAF of 41.35%, 10 cases (9.35%) had the *R625* mutation with the median VAF of 37.10%, 6 cases (5.61%) had the *H662* mutation with the median VAF of 21.00%, and 5 cases (4.67%) had the *E622* mutation with the median VAF of 35.00% (Figure 1b and Table 1). Patients with *E622* and *H662* mutations showed lower VAF compared to those with *non‐E622* and *non‐H662* mutations (median, 22.35% vs. 40.20%, *p* = 0.059), and *SF3B1* ^*H662*^ patients showed significantly lower VAF (median, 21.00% vs. 40.00%, *p* = 0.034, Figure S1). Two patients had double mutation spots. One case had both *K700E* and *E622* mutations, and the other had *K700E* and *R939H* mutations. The remaining hotspots were *E592K*, *A744P*, *G740E*, *L638F*, *G751V*, and *A1282E* (7/107, 6.54%) (Figure 1b). In our cohort, the median hemoglobin was 71.8 g/L in patients with the *SF3B1* mutation. Of the total patients, 51.4% showed moderate anemia, 28.0% showed severe anemia, and 11.2% showed mild anemia. The median hemoglobin was 76.0 g/L in patients with *E622* and *H662* mutations compared to those without these mutations (median, 76.0 g/L vs. 68.5 g/L, *p* = 0.059). *SF3B1* ^*K666*^ patients showed significantly lower platelets compared to those with *non‐K666* mutations (median, 78.0 × 10^9^/L vs. 127.5 × 10^9^/L, *p* = 0.032, Figure 1d), whereas *SF3B1* ^*R625*^ patients showed significantly higher platelets compared to those with *non‐R625* mutations (median, 189.0 × 10^9^/L vs. 111.5 × 10^9^/L, *p* = 0.020, Figure 1d). The platelet count had no statistical significance in patients with *E622* and *H662* mutations compared to those with other subtype mutations. There was no discernible difference in the absolute neutrophil count within the five groups with varying subtype mutations (Figure 1e). The percentage of BM blasts was lower in *SF3B1* ^*mut*^ patients with *E622* and *H662* hotspots, although without statistical significance (median, 0.50% in *E622* and *H662* vs. 1.35% in *non‐E622* and *non‐H662*, *p* = 0.060). In our cohort, 75.0% of patients with the *SF3B1* mutation had a RS percentage higher than 5%. Of these patients, 90.5% had a percentage of RS greater than 15%. Of *SF3B1* ^*mut*^ patients with the *K700E* mutation, 88.6% had an RS percentage higher than 5%, compared to 71.4% of *SF3B1* ^*mut*^ patients with the *R625* mutation, 66.7% of *SF3B1* ^*mut*^ patients with *E622*, 63.2% of *SF3B1* ^*mut*^ patients with the *K666* mutation, and 25.0% of *SF3B1* ^*mut*^ patients with the *H662* mutation. The percentage of RS was significantly lower in *SF3B1* ^*mut*^ patients with the *K666* mutation compared to those with *non‐K666* mutations (median, 16.8% vs. 36.0%, *p* = 0.021), while significantly higher in *SF3B1* ^*mut*^ patients with the *K700E* mutation compared to those with *non‐K700E* mutations (median, 39.0% vs. 18.0%, *p* = 0.013). In our cohort, 30.8% of *SF3B1* ^*mut*^ patients had a serum ferritin level above 1000 ng/mL. The median serum ferritin was significantly lower in *SF3B1* ^*mut*^ patients with the *K666* mutation compared to those with *non‐K666* mutations (median, 497.1 ng/mL vs. 874.0 ng/mL, *p* = 0.022). A total of 47.7% of patients had serum erythropoietin above 500 mIU/mL.

**TABLE 1:** Clinical characteristics of mutation sites in *SF3B1* ^*mut*^ MDS.

Characteristics	*E622*, *n* = 5	*R625*, *n* = 10	*H662*, *n* = 6	*K700E*, *n* = 51	*K666*, *n* = 26	*p*
HGB						
Median (g/L)	69.0	69.0	76.0	68.0	69.0	0.342
Range (g/L)	63.0 to 116.5	63.0 to 72.0	70.5 to 105.3	55.5 to 77.3	57.0 to 92.0	
PLT						
Median (10^9^/L)	128.0	189.0	160.5	112.0	78.0	**0.050**
Range (10^9^/L)	23.8 to 190.0	122.0 to 315.5	91.3 to 260.8	56.5 to 183.3	45.0 to 140.0	
ANC						
Median (10^9^/L)	0.95	1.40	2.60	1.70	1.05	0.208
Range (10^9^/L)	0.65 to 1.78	0.90 to 4.15	1.95 to 3.65	0.88 to 2.90	0.70 to 3.13	
RS						
Median (%)	22.0	42.0	0.0	39.0	16.8	0.070
Range (%)	0.0 to —	4.0 to 50.0	0.0 to 55.5	16.0 to 58.0	0.0 to 43.5	
BM blasts						
Median (%)	0.50	1.00	0.45	1.35	1.90	0.404
Range (%)	0.20 to 6.70	0.60 to 4.47	0.05 to 6.45	0.60 to 3.50	0.60 to 6.75	
CD4						
Median (%)	29.58	31.34	37.33	31.71	33.98	0.595
Range (%)	29.04 to 38.25	23.95 to 37.02	32.09 to 41.00	25.89 to 37.28	26.58 to 37.54	
CD8						
Mean (%)	37.08	36.12	33.68	37.97	38.54	0.948
Range (%)	16.00 to 51.00	24.80 to 52.30	25.00 to 42.83	13.47 to 75.48	10.00 to 65.69	
CD4/CD8						
Median	0.86	0.77	1.14	0.92	0.83	0.721
Range	0.66 to 1.57	0.65 to 1.31	0.81 to 1.53	0.61 to 1.12	0.67 to 1.05	
Th1						
Median (%)	10.00	22.40	26.79	22.42	17.21	0.862
Range (%)	3.75 to 43.18	12.19 to 37.99	7.50 to 32.25	13.17 to 33.70	12.69 to 34.07	
Th1/Th2						
Median	49.0	36.4	49.1	44.8	19.0	0.162
Range	21.5 to 103.9	13.6 to 99.8	35.0 to 134.2	21.9 to 94.1	11.8 to 56.4	
Tc1						
Mean (%)	39.42	45.10	40.77	48.55	56.51	0.510
Range (%)	4.60 to 76.10	16.42 to 70.00	13.50 to 63.78	2.20 to 88.19	23.00 to 93.95	
Tc1/Tc2						
Median	149.5	74.9	151.6	130.0	123.6	0.560
Range	102.5 to 242.1	37.3 to 150.9	74.0 to 365.0	75.5 to 272.0	72.9 to 218.5	
NK						
Median (%)	18.45	24.10	14.00	18.30	8.41	**0.032**
Range (%)	8.75 to 40.23	14.58 to 35.17	9.89 to 20.82	9.54 to 24.32	3.66 to 20.56	
VAF						
Median (%)	35.00	37.10	21.00	39.65	41.35	0.299
Range (%)	12.10 to 51.30	32.68 to 44.83	16.25 to 34.10	29.90 to 44.85	28.15 to 47.15	
Number of mutant genes						
1, *n* (%)	1 (20.0)	0 (0.0)	1 (16.7)	7 (13.7)	6 (23.1)	0.468
2, *n* (%)	3 (60.0)	3 (30.0)	2 (33.3)	17 (33.3)	5 (19.2)	
≥ 3, *n* (%)	1 (20.0)	7 (70.0)	3 (50.0)	27 (52.9)	15 (57.7)	
TET2						
TET2, *n* (%)	2 (40.0)	5 (50.0)	0 (0.0)	8 (15.7)	5 (19.2)	0.068
Non‐TET2, *n* (%)	3 (60.0)	5 (50.0)	6 (100.0)	43 (84.3)	21 (80.8)	
ASXL1						
ASXL1, *n* (%)	0 (0.0)	0 (0.0)	4 (66.7)	9 (17.6)	4 (15.4)	**0.027**
Non‐ASXL1, *n* (%)	5 (100.0)	10 (100.0)	2 (33.3)	42 (82.4)	22 (84.6)	
TP53						
TP53, *n* (%)	1 (20.0)	1 (10.0)	0 (0.0)	9 (17.6)	2 (7.7)	0.653
Non‐TP53, *n* (%)	4 (80.0)	9 (90.0)	6 (100.0)	42 (82.4)	24 (92.3)	
DNMT3A						
DNMT3A, *n* (%)	0 (0.0)	2 (20.0)	0 (0.0)	7 (13.7)	3 (11.5)	0.880
Non‐ DNMT3A, *n* (%)	5 (100.0)	8 (80.0)	6 (100.0)	44 (86.3)	23 (88.5)	
RUNX1						
RUNX1, *n* (%)	0 (0.0)	1 (10.0)	0 (0.0)	5 (9.8)	1 (3.8)	0.839
Non‐ RUNX1, *n* (%)	5 (100.0)	9 (90.0)	6 (100.0)	46 (90.2)	25 (96.2)	
STAG2						
STAG2, *n* (%)	0 (0.0)	0 (0.0)	1 (16.7)	1 (2.0)	2 (7.7)	0.270
Non‐ STAG2, *n* (%)	5 (100.0)	10 (100.0)	5 (83.3)	50 (98.0)	24 (92.3)	
EZH2						
EZH2, *n* (%)	0 (0.0)	0 (0.0)	1 (16.7)	3 (5.9)	1 (3.8)	0.615
Non‐EZH2, *n* (%)	5 (100.0)	10 (100.0)	5 (83.3)	48 (94.1)	25 (96.2)	
Status						
AML transformation, *n* (%)	0 (0.0)	1 (11.1)	1 (16.7)	7 (14.3)	8 (32.0)	0.324
Non‐AML transformation, *n* (%)	5 (100.0)	8 (88.9)	5 (83.3)	42 (85.7)	17 (68.0)	

*Note:* Bold part indicates meaningful statistical results.

Abbreviations: AML, acute myeloid leukemia; ANC, absolute neutrophil count; BM, bone marrow; HGB, hemoglobin; PLT, platelet; RS, ringed sideroblasts; VAF, variant allele frequency.

The serum erythropoietin did not reach the statistical difference between patients with distinct subtype mutations (Table S1). Among the IPSS‐R categories, *SF3B1* ^*K666*^ patients showed a higher percentage in the higher risk group compared to those with *non‐K666* mutations (36.8% vs. 20.3%, *p* = 0.024). Conversely, almost all of the patients with the *E622* mutation were in the lower risk group. Regarding the 2016 WHO categories, *SF3B1* ^*mut*^ patients were both enriched in WHO categories with MDS‐RS. MDS with isolated del (5q) was only found in patients with the *R625* mutation (Table S1). Further analysis of the above characteristics showed no differences in the subgroup of patients with a normal karyotype (Table S2).

### 3.2 Immune Characteristics of Mutation Spots in *SF3B1* ^*mut*^ MDS

**FIGURE 2:**
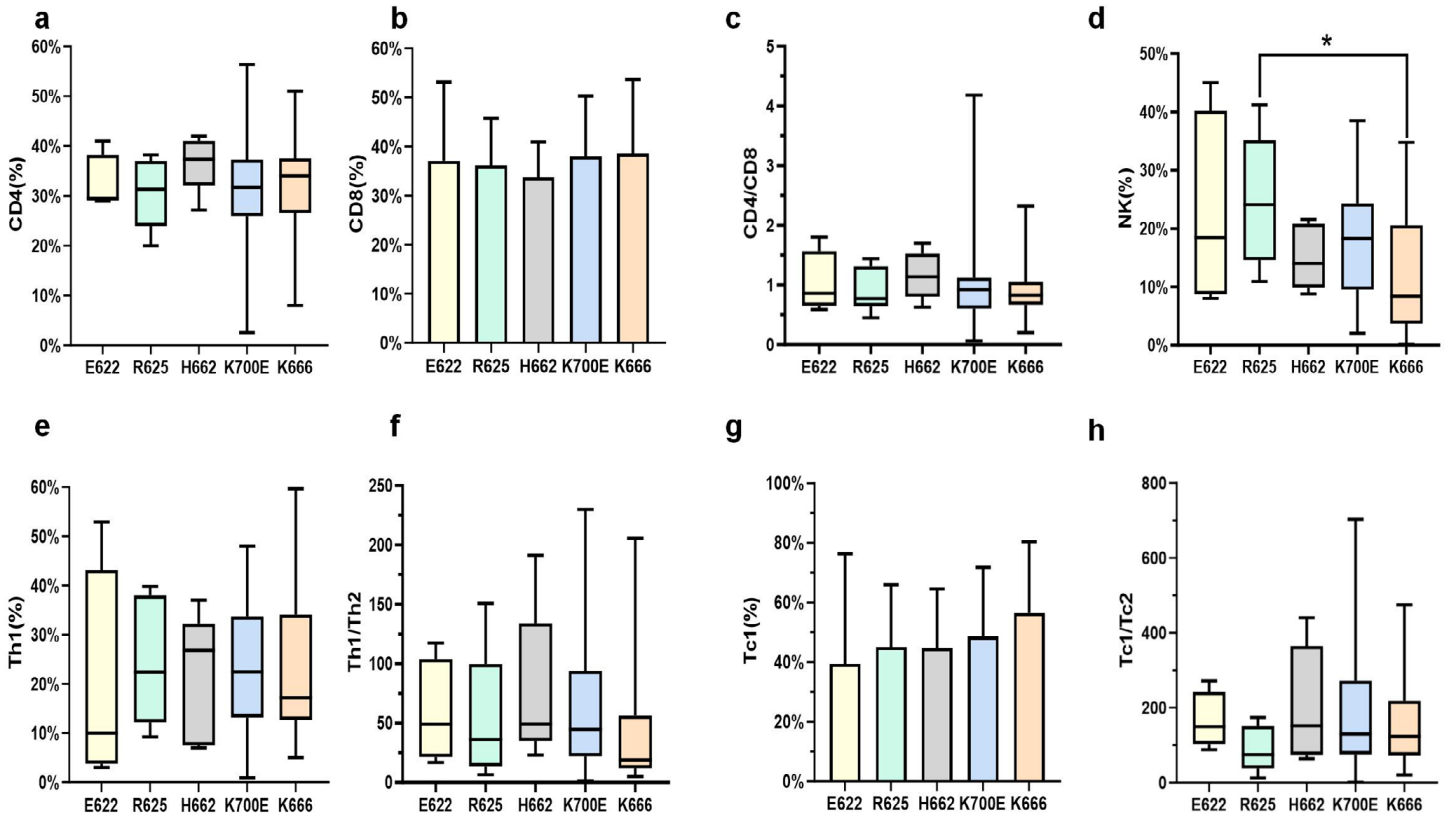
T cell subsets and NK cells in the BM of mutation spots of *SF3B1* ^*mut*^ MDS. (a) The difference in the CD4^+^ T cell percentage in *SF3B1* ^*mut*^ MDS patients with distinct mutation spots. (b) The difference in the CD8^+^ T cell percentage in *SF3B1* ^*mut*^ MDS patients with distinct mutation spots. **(c)** The difference in the CD4/CD8 ratio in *SF3B1* ^*mut*^ MDS patients with distinct mutation spots. (d) The difference in NK cell percentage in *SF3B1* ^*mut*^ MDS patients with distinct mutation spots. (e) The difference in Th1 cell percentage in *SF3B1* ^*mut*^ MDS patients with distinct mutation spots. (f) The difference in the Th1/Th2 ratio in *SF3B1* ^*mut*^ MDS patients with distinct mutation spots. (g) The difference in Tc1 cell percentage in *SF3B1* ^*mut*^ MDS patients with distinct mutation spots. (h) The difference in the Tc1/Tc2 ratio in *SF3B1* ^*mut*^ MDS patients with distinct mutation spots. *p = 0.028.

The analysis of immune characteristics of *SF3B1* ^*mut*^ patients mainly included CD4^+^ T cells, CD8^+^ T cells, CD4/CD8 ratio, Th1 cells, Th1/Th2 ratio, Tc1 cells, Tc1/Tc2 ratio, and NK cells in the BM. *SF3B1* ^*R625*^ patients showed a significantly higher BM NK cell percentage (median: 24.10% vs. 14.67%, *p* = 0.025) compared to those with *non‐R625* mutations, whereas *SF3B1* ^*K666*^ patients showed a significantly lower BM NK cell percentage compared to those with *non‐K666* mutations (median, 8.41% vs. 18.59%, *p* = 0.001). BM NK cell percentage was significantly higher in *SF3B1* ^*mut*^ patients with the *R625* mutation compared to those with the *K666* mutation (median, 24.10% vs. 8.41%, *p* = 0.028, Figure 2d). In addition, *SF3B1* ^*mut*^ patients with *K666* mutations showed a significantly lower Th1/Th2 ratio compared to those with *non‐K666* mutations (median: 19.0% vs. 45.6%, *p* = 0.018, Figure 2f).

### 3.3 Concomitant Gene Mutations and Cytogenetic Changes in *SF3B1* ^*mut*^ MDS

The most frequently mutant genes co‐occurring with *SF3B1* included *TET2* (20.6%).

**FIGURE 3:**
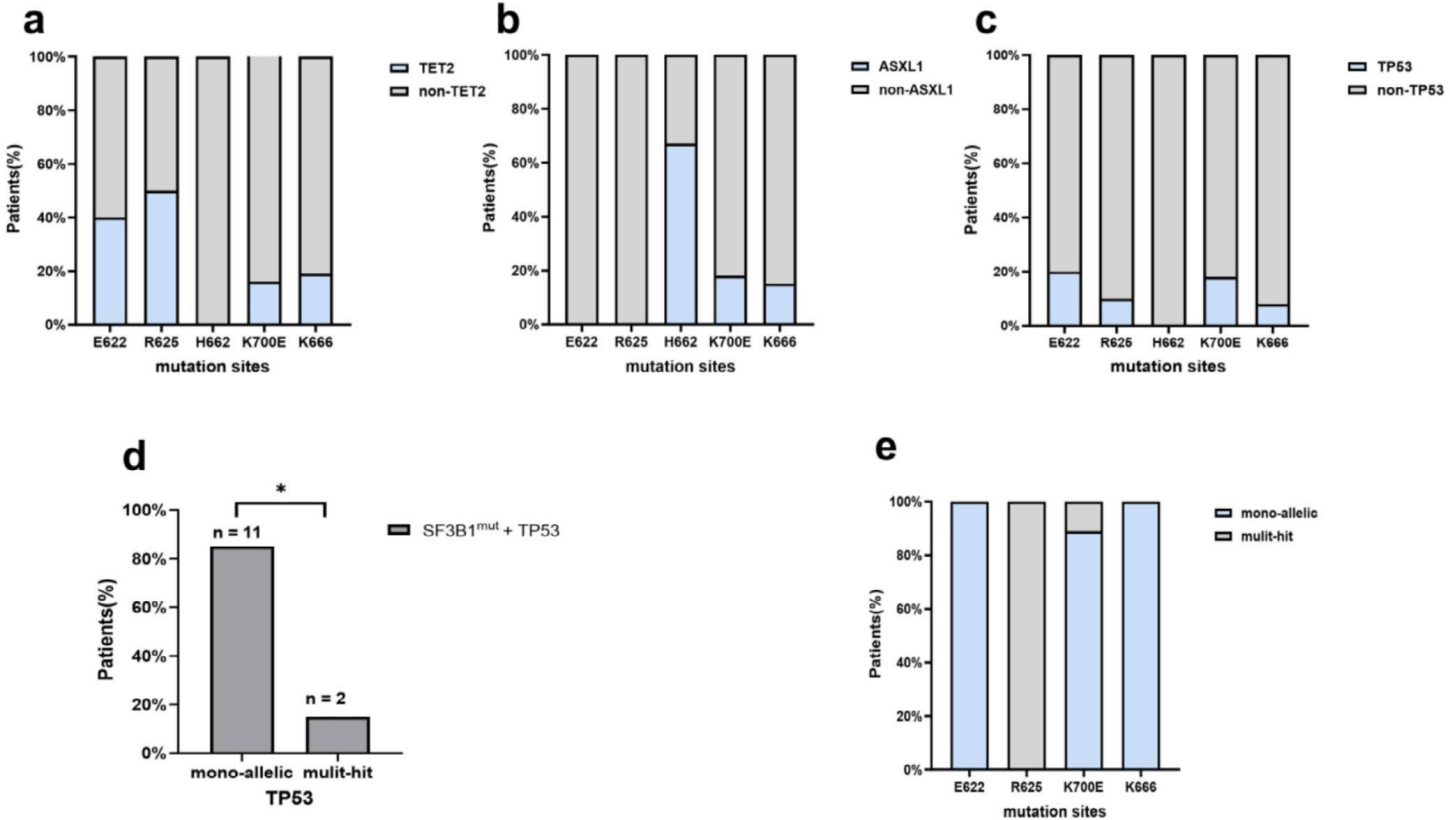
Concomitant gene mutations of mutation spots of *SF3B1* ^*mut*^ MDS. (a) The proportion of *TET2* mutation in *SF3B1* ^*mut*^ MDS patients with distinct mutation spots. (b) The proportion of *ASXL1* mutation in *SF3B1* ^*mut*^ MDS patients with distinct mutation spots. (c) The proportion of *TP53* mutation in *SF3B1* ^*mut*^ MDS patients with distinct mutation spots. (d) The *TP53* allelic state in *SF3B1* ^*mut*^ MDS (e) The *TP53* allelic state in *SF3B1* ^*mut*^ MDS patients with distinct mutation spots. *p = 0.013.

**FIGURE 4:**
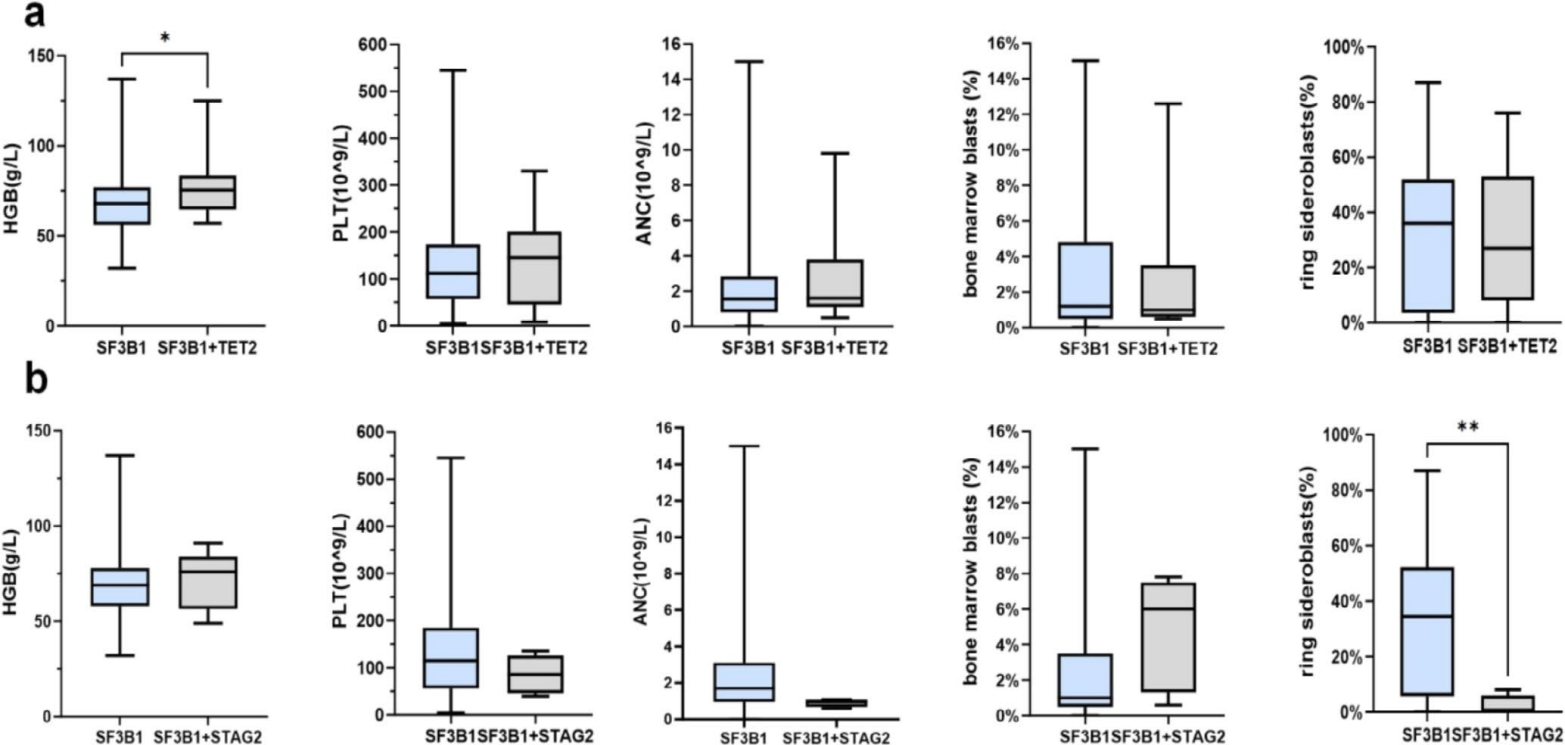
Clinical characteristics of concomitant gene mutations in *SF3B1* ^*mut*^ MDS. (a) The clinical characteristics of *SF3B1* ^*mut*^ MDS patients with the *TET2* mutation. (b) The clinical characteristics of *SF3B1* ^*mut*^ MDS patients with the *STAG2* mutation. *p = 0.050; **p = 0.010.

*ASXL1* (19.6%), *TP53* (12.1%), *DNMT3A* (11.2%), *RUNX1* (6.5%), *EZH2* (4.7%), and *STAG2* (4.7%). *TP53* mutations were divided into mono‐hit and multi‐hit according to the WHO 2022 classification for MDS. In our cohort of 107 cases with *SF3B1* mutations, *TP53* mutations were detected in 13 (12.1%) patients. Notably, most of the mutations in *TP53* (11/13, 84.6%) were mono‐hit (Figure 3d). In *SF3B1* ^*mut*^ patients with the *H662* hotspot, *ASXL1* (4/6, 66.7%) was the most frequently co‐mutated gene, whereas *TET2*, *TP53*, *DNMT3A*, and *RUNX1* mutations were not observed (Figure 3b). *TET2* (5/10, 50%) was the most frequently co‐mutant gene in patients with the *R625* mutation, whereas *ASXL1*, *STAG2*, and *EZH2* mutations were not observed (Figure 3a). Further analysis showed *TET2* was also the common co‐mutant gene in the subgroup of *SF3B1* ^*R625*^ patients with normal karyotype (Table S2). Three patients had double‐splicing gene mutations, which were the co‐occurrence of the *SF3B1* and *U2AF1* mutation, the *SF3B1* mutation and *ZRSR2* mutation, and the *SF3B*1 mutation and *SRSF2* mutation. Interestingly, the percentage of RS was significantly lower in *SF3B1* ^*mut*^ MDS patients with *STAG2* mutation (median, 0.0% vs. 34.5%, *p* = 0.010, Figure 4b). Of patients with the co‐occurrence of the *SF3B1* mutation with the *STAG2* mutation, 75% showed no percentage of RS. However, there were no differences in VAF between patients with or without specific co‐mutations (Figure S4).

Cytogenetic analysis showed 43.9% of patients with the *SF3B1* mutation had the normal karyotype, which had no statistical difference in *SF3B1* ^*mut*^ patients with specific mutation spots. The t (3q) and inv (3q) were observed in two *SF3B1* ^*mut*^ patients with the *K700E* mutation. In addition, t (3q) was also observed in a patient with *K700E* and *E622* double mutation spots. A patient with the *K666* mutation had a loss of chromosome 7 (Table S1). Of *SF3B1* ^*mut*^ patients, 6.5% had the complex karyotype. Further analysis showed no patients had the complex karyotype in *SF3B1* ^*mut*^ patients with the *E622* mutation (Figure 1h). In *SF3B1* ^*mut*^ patients with the *K666* mutation, the percentage of complex karyotype was significantly lower compared to the percentage of normal karyotype (3.8% vs. 50.0%, *p =* 0.001).

### 3.4 Prognosis of Patients With *SF3B1* ^*mut*^ MDS

**FIGURE 5:**
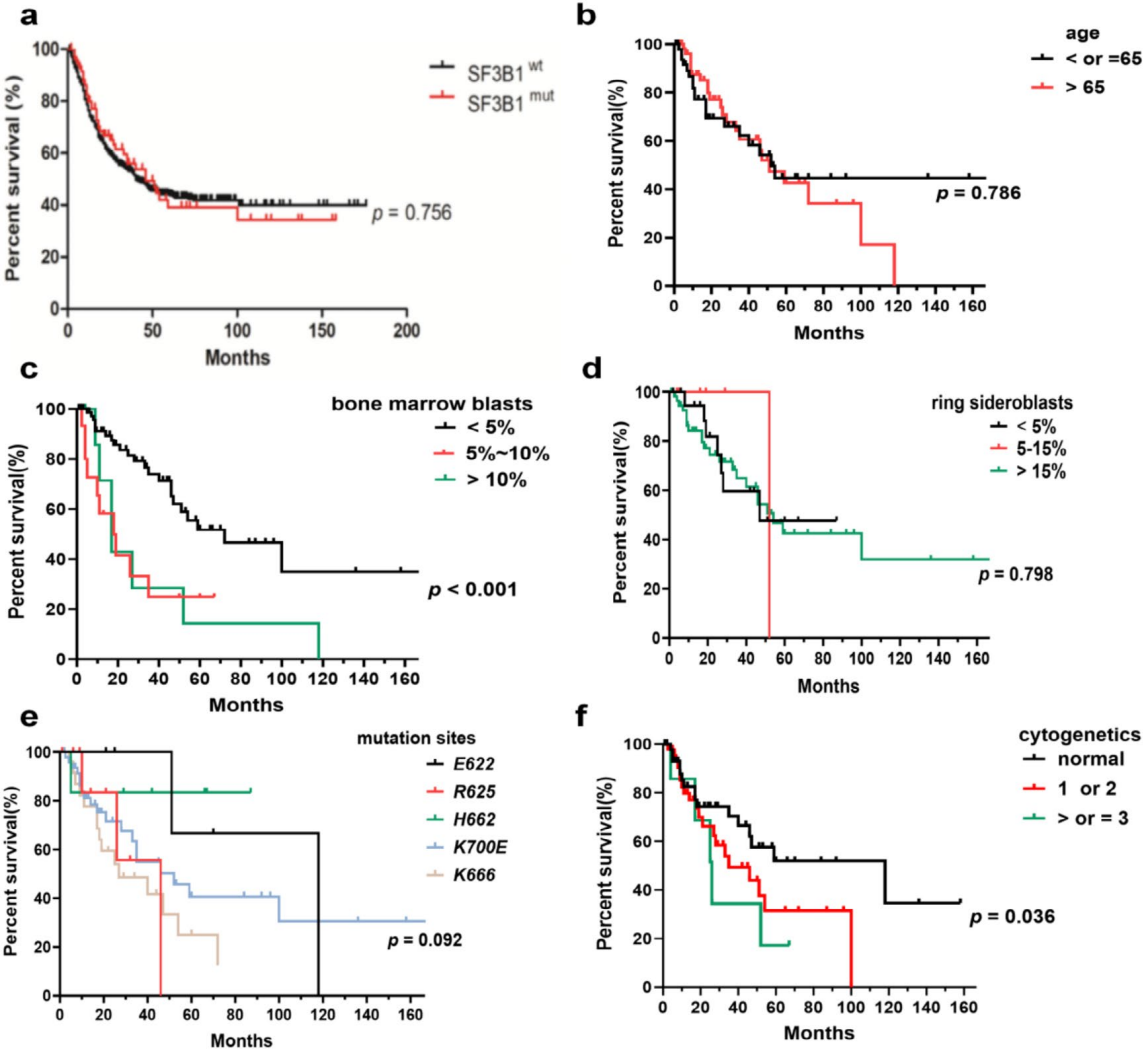
Kaplan–Meier curves for OS in *SF3B1* ^*mut*^ MDS patients. (a) Kaplan–Meier curve for OS between *SF3B1* ^*mut*^ MDS patients and *SF3B1* ^*wt*^ MDS patients. (b) Kaplan–Meier curve for OS in *SF3B1* ^*mut*^ MDS patients between the ages of under 65 and over 65. (c) Kaplan–Meier curve for OS in *SF3B1* ^*mut*^ MDS patients with different percentages of BM blasts. (d) Kaplan–Meier curve for OS in *SF3B1* ^*mut*^ MDS patients with different percentages of RS. (e) Kaplan–Meier curve for OS in *SF3B1* ^*mut*^ MDS patients with distinct mutation spots. (f) Kaplan–Meier curve for OS of *SF3B1* ^*mut*^ MDS patients with distinct karyotypes.

**FIGURE 6:**
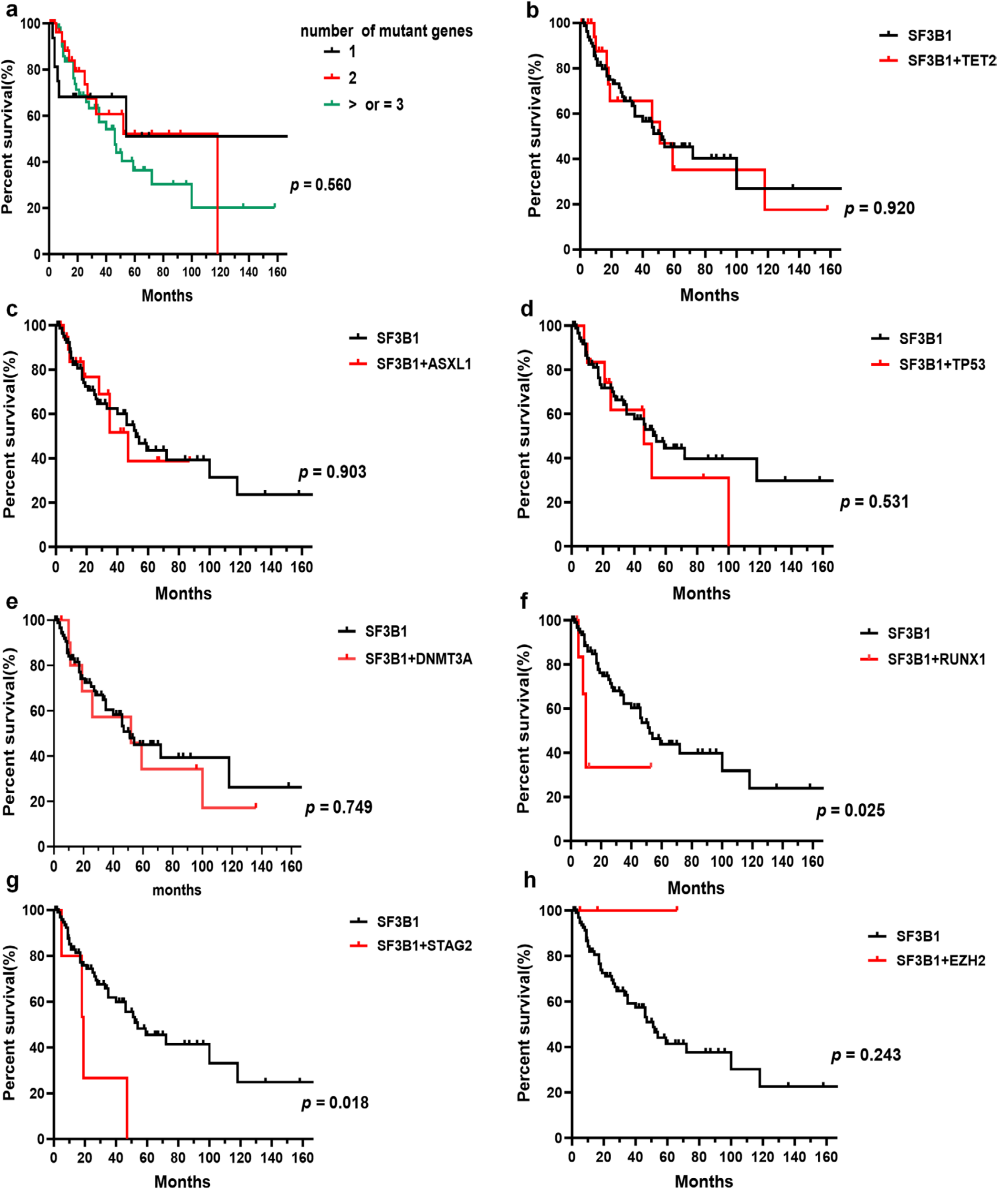
Kaplan–Meier curves for OS in *SF3B1* ^*mut*^ MDS patients. (a) Kaplan–Meier curve for OS in *SF3B1* ^*mut*^ MDS patients with different numbers of mutant genes. (b) Kaplan–Meier curve for OS between *SF3B1* ^*mut*^ MDS patients with *TET2* mutation and *non‐TET2* mutation. (c) Kaplan–Meier curve for OS between *SF3B1* ^*mut*^ MDS patients with *ASXL1* mutation and *non*‐*ASXL1* mutation. (d) Kaplan–Meier curve for OS between *SF3B1* ^*mut*^ MDS patients with *TP53* mutation and *non‐TP53* mutation. (e) Kaplan–Meier curve for OS between *SF3B1* ^*mut*^ MDS patients with *DNMT3A* mutation and *non*‐*DNMT3A* mutation. (f) Kaplan–Meier curve for OS between *SF3B1* ^*mut*^ MDS patients with *RUNX1* mutation and *non*‐*RUNX1* mutation. (g) Kaplan–Meier curve for OS between *SF3B1* ^*mut*^ MDS patients with *STAG2* mutation and *non*‐*STAG2* mutation. (h) Kaplan–Meier curve for OS between *SF3B1* ^*mut*^ MDS patients with *EZH2* mutation and *non*‐*EZH2* mutation.

We analyzed the prognostic impact of gene mutations and other risk factors on OS in *SF3B1* ^*mut*^ MDS. The median OS in *SF3B1* ^*mut*^ patients showed no statistical difference compared to *SF3B1* ^*wt*^ patients (Figure 5a). The median OS of patients between the ages of under 65 and over 65 was similar (Figure 5b). The median OS of patients with BM blasts less than 5% was 72 months, which was longer than those with BM blasts more than 5% (*p* = 0.0004, Figure 5c). The median OS of patients with RS below 5%, between 5% and 15%, and above 15% was 47, 52, and 54 months, respectively, which showed no statistical difference (Figure 5d). Patients with *E622* and *H662* mutations had longer OS than those with other subtype mutations, which was significantly longer than patients with the *R625* mutation (median OS, 118 months in *E622* and *H662* vs. 46 months in *R625*, *p* = 0.045) and the *K666* mutation (median OS, 118 months in E622 and H662 vs. 27 months in K666, *p* = 0.010). The S*F3B1‐K666* mutation showed an inferior impact on the prognosis of patients compared to non*‐K666* mutations (median OS, 27 months vs. 52 months, *p* = 0.043). The prognostic impact of karyotypes on OS showed statistical significance (median OS, 118 months for patients with normal karyotypes vs. 35 months for patients with 1 or 2 abnormal karyotypes vs. 26 months for patients with complex karyotypes, *p* = 0.036, Figure 5f). Of *SF3B1* ^*mut*^ patients with the *K666* mutation, 32.0% progressed to AML, compared to 16.7% of *SF3B1* ^*mut*^ MDS patients with the *H662* mutation, 14.3% of *SF3B1* ^*mut*^ patients with *K700E*, and 11.1% of *SF3B1* ^*mut*^ patients with the *R625* mutation. No patients with the *E622* mutation progressed to AML (Table 1). Within all *SF3B1* ^*mut*^ patients, the number of mutant genes showed no significant impact on OS (Figure 6a). Compared to *SF3B1* ^*mut*^ with *non*‐*RUNX1* and *non‐STAG2* co‐mutations, the median OS of patients with *RUNX1* (median OS, 10 months vs. 52 months, *p* = 0.025, Figure 6f) and *STAG2* co‐mutations (median OS, 19 months vs. 54 months, *p* = 0.018, Figure 6 g) was worse. The co‐mutations of *TET2*, *ASXL1*, *TP53*, *DNMT3A*, and *EZH2* showed no discernible effects on OS (Figure 6b–e,h). Within a single‐variable survival analysis, the BM blasts (HR 1.912, *p* < 0.001), cellular genetics (HR 1.627, *p* = 0.039), *K666* mutation (HR 2.037, *p* = 0.030), *RUNX1* co‐mutation (HR 3.106, *p* = 0.034), and *STAG2* co‐mutation (HR 3.262, *p* = 0.027) were unfavorable factors of prognosis (Table 2). In multivariate survival analysis, the BM blasts (HR 1.661, *p =* 0.017), *K666* mutation (HR 2.094, *p* = 0.050), and *RUNX1* co‐mutation (HR 4.445, *p* = 0.021) were independent prognostic factors (Table 2), but cytogenetics did not reveal significant differences (HR 1.539, *p* = 0.091, Table 2).

**TABLE 2:** The analysis for the overall survival of *SF3B1* ^*mut*^ MDS.

Risk factors	HR	95% CI	*p*
**Univariate analysis**			
**Sex**	0.622	0.327–1.184	0.148
Age (≤ 65 vs. > 65)	1.030	0.560–1.895	0.923
**BM blasts** (%) (< 5 vs. 5–10 vs. > 10)	1.912	1.309–2.793	**< 0.001**
**RS** (%) (< 5 vs. 5–15 vs. > 15)	1.058	0.687–1.628	0.799
**Number of mutant genes** (1 vs. 2 vs. ≥ 3)	1.135	0.739–1.742	0.563
**Mutation site**			
*E622*	0.534	0.128–2.228	0.390
*R625*	1.348	0.410–4.429	0.623
*H662*	0.258	0.035–1.881	0.181
*K700E*	0.967	0.522–1.791	0.916
*K666*	2.037	1.072–3.872	**0.030**
**Co‐mutation**			
*TET2* (*SF3B1* vs. *SF3B1* + *TET2*)	1.039	0.493–2.189	0.920
*ASXL1* (*SF3B1* vs. *SF3B1* + *ASXL1*)	1.049	0.482–2.282	0.904
*TP53* (*SF3B1* vs. *SF3B1* + *TP53*)	1.294	0.573–2.924	0.535
*DNMT3A* (*SF3B1* vs. *SF3B1* + *DNMT3A*)	1.143	0.502–2.601	0.750
*RUNX1* (*SF3B1* vs. *SF3B1* + *RUNX1*)	3.106	1.087–8.876	**0.034**
*STAG2* (*SF3B*1 vs. *SF3B1* + *STAG2*)	3.262	1.147–9.277	**0.027**
*EZH2* (*SF3B1* vs. *SF3B1* + *EZH2*)	0.047	0.000–115.304	0.443
**Cytogenetics** Normal vs. 1 or 2 vs. ≥ 3	1.627	1.024–2.583	**0.039**
**Multivariate analysis**			
**BM blasts** (%) (< 5 vs. 5–10 vs. > 10)	1.661	1.093–2.524	**0.017**
**Mutation site**			
*K666*	2.094	1.001–4.381	**0.050**
**Co‐mutation**			
*RUNX1* (*SF3B1* vs. *SF3B1* + *RUNX1*)	4.445	1.254–15.756	**0.021**
*STAG2* (*SF3B1* vs. *SF3B1* + *STAG2*)	2.479	0.818–7.519	0.109
**Cytogenetics** Normal vs. 1 or 2 vs. ≥ 3	1.539	0.943–2.535	0.091

*Note*: Bold part indicates meaningful statistical results.

Abbreviations: BM, bone marrow; CI, confidence interval; HR, Hazard ratio; RS, ringed sideroblasts.

## 4 Discussion

The International Working Group for the Prognosis of MDS has proposed *SF3B1* ^*mut*^ MDS as a distinct disease subtype [1]. In the 5th edition of the classification of hematolymphoid tumors (WHO‐HAEM5), MDS with low blasts and *SF3B1* mutation was defined as a new subtype [6]. *SF3B1* ^*mut*^ patients have many similar characteristics, but specific subtypes may show distinct clinical features and prognosis. With further study about the mechanisms of MDS, besides cellular and molecular genetics, immunological therapy also provides a new possibility for *SF3B1* ^*mut*^ MDS. Therefore, the identification of different mutant subtypes is critical for risk stratification and therapeutic decision‐making. In addition, the studies of MDS with *SF3B1* mutations should be individualized and comprehensive. To better understand the clinical characteristics and prognosis of *SF3B1* ^*mut*^ MDS, our research primarily focused on mutant subtypes.

Multiple researches have shown that *SF3B1* ^*mut*^ patients usually have a lower probability of conversion to AML and are associated with a positive prognosis. The most common clinical feature is anemia. In our cohort, only five patients with the *SF3B1* mutation showed no anemia. *SF3B1* mutations predominantly involved *K700* and, to a lesser extent, *K666*, *H662*, and *E622* [2]. *K700E* was the most frequent mutation spot in 47.66% of cases in our cohort, similar to what was noted in previous studies. *K666* and *R625* were observed in 24.30% and 9.35%, respectively. Two less frequent mutation subtypes accounted for 5.61% of *H662* and 4.67% of *E622*. Kanagal‐Shamanna et al. discovered that only patients with the *K700E* mutation had a good prognosis [11]. A previous study observed that the *E622* hotspot was enriched early in the disease and presumed that the *E622* hotspot may contribute to indolent MDS [12]. In our study, we further analyzed the clinical features of patients with the *E622*, *R625*, and *H662* mutations. We did not observe complex karyotypes and AML transformation in patients with the *E622* mutation. *SF3B1* ^*mut*^ patients with the *E622* and *H622* mutations had higher hemoglobin and a lower percentage of BM blasts. However, when compared to patients with other subtype mutations, the above result did not reach statistical significance due to insufficient samples. In our cohort, 67.3% of the patients had VAF higher than 30%. *SF3B1* ^*E622*^ and *SF3B1* ^*H622*^ patients showed lower VAF compared to *SF3B1* ^*non‐E622*^ and *SF3B1* ^*non‐H622*^ patients, and VAF was significantly lower in *SF3B1* ^*H662*^ patients. Further analysis showed that the median OS was longer in patients with *E622* and *H662* mutations, which was significantly longer than in patients with *R625* and *K666* mutations. Although the independent positive prognostic value of the *E622* and *H662* hotspots did not reach statistical significance, the *E622* and *H662* mutations may be associated with a positive prognosis, which may need to be confirmed by a larger cohort. Patients with the *R625* mutation were less likely to show thrombocytopenia, but we did not observe distinct effects on prognosis. Similar to previous findings [12], we observed that *SF3B1* ^*K666*^ patients more often showed thrombocytopenia and higher IPSS‐R scores. Patients with the *K666* mutation were more likely to develop AML. Although most of the patients in this cohort had the normal karyotype, further analysis of the clinical characteristics in this subgroup with specific mutation sites showed no significant differences. Interestingly, the lower percentage of complex karyotype did not change the poor prognosis in MDS with the *K666* mutation. In our cohort, the *SF3B1* mutation involving *K666* independently predicted overall survival in MDS. Interestingly, we observed two patients with double mutation spots. One patient with *K700E* and *E622* double mutation spots, diagnosed with MDS‐RS and classified as an intermediate‐risk group, showed severe anemia and a poor chromosome karyotype. Another patient with *K700E* and *R939H* mutations, diagnosed with MDS‐RS and classified as a low‐risk group, showed moderate anemia and a good chromosome karyotype.

Previous study has observed that the *SF3B1* mutation may be an initiating genetic event [13]. In most patients, *SF3B1* may occur as the first event, whereas it may appear to be secondary to other mutations in a minority of cases [14]. In our cohort, four patients did not have the *SF3B1* mutation when they were initially diagnosed with MDS, followed by *K700E* mutations, and showed different clinical features. One showed severe anemia and progressed to death; the other showed moderate anemia; the other showed severe anemia, thrombocytopenia, and leukopenia; and the last one showed thrombocytopenia and leukopenia. These interesting findings allow us to further explore potential mechanisms of splicing mutation in MDS. Our inability to reach statistical significance could be due to insufficient samples. Therefore, research in larger cohorts is warranted.

The *SF3B1* mutation had a significant correlation with the percentage of RS [15]. RS is present in about 80% of MDS with *SF3B1* mutations. In addition, MDS with low blasts and *SF3B1* (MDS‐*SF3B1*), a distinct disease type, includes over 90% of MDS with the percentage of RS ≥ 5 [1]. In our cohort, 75.0% of patients with the *SF3B1* mutation had a RS percentage higher than 5%. However, the exact mechanism of the formation of RS in *SF3B1* ^*mut*^ is complex and unclear. Previous studies have found the *SF3B1* mutation may be associated with abnormal gene splicing related to iron metabolism [4, 5, 16]. The disorder of gene expression involved in iron metabolism, such as *A* *BCB7*, *ALAS2*, *GATA‐*1, and *MAP3K7* genes, may be associated with the formation of RS. However, the prognostic value of RS in MDS is limited [15]. In our cohort, the percentage of RS was significantly lower in *SF3B1* ^*mut*^ patients with the *K666* mutation compared to those with *non‐K666* mutations, while significantly higher in *SF3B1* ^*mut*^ patients with the *K700E* mutation compared to those with *non‐K700E* mutations. We speculate that mutational subtypes in *SF3B1* may cause different gene splicing in iron metabolism, which may lead to changes in the percentage of RS. The *STAG2* mutation, as the most frequently mutated cohesin mutation in myeloid malignancies, is essential for chromatin structure and replication [17]. The *SF3B1* mutation and *STAG2* tend not to occur simultaneously [13]. In our cohort, only 4.7% of *SF3B1* ^*mut*^ patients had *STAG2* co‐mutation. Interestingly, we observed a significantly lower percentage of RS in these patients, and three patients had no percentage of RS, which may need to be further studied by a larger cohort due to the insufficient cases with *SF3B1* and *STAG2* co‐mutation.

An excess of 1000 ng/mL of serum ferritin signifies iron overload [18, 19], which may inhibit erythropoiesis by preventing the proliferation of erythrocyte progenitor cells [20, 21]. In addition, when serum erythropoietin exceeds 500 mIU/mL, the reactivity of the erythropoiesis‐stimulating drugs (ESA) may decrease [22, 23, 24], and the high serum erythropoietin level is associated with lower hemoglobin. In our cohort, only 30.8% of *SF3B1* ^*mut*^ patients had a serum ferritin level above 1000 ng/mL, whereas 47.7% of patients had a serum erythropoietin level above 500 mIU/mL. We also compared the differences in serum ferritin and serum erythropoietin in *SF3B1* ^*mut*^ patients with distinct mutation spots. However, we observed no significant difference, which could be due to the limited number of cases and the heterogeneity of our cohort.

*SF3B1* ^*mut*^ patients with complex mutational status (at least two additional mutations) are associated with a poor prognosis [1, 25]. More than 40% of patients with MDS have at least two mutations, and the co‐occurrence of particular genes may change the effect on the prognosis of the *SF3B1* mutation. IPSS‐R considers hemoglobin, absolute neutrophil count, platelet count, percentage of BM blasts, and cytogenetics, but gene mutations are not used in the risk stratification of patients with MDS. Based on IPSS‐R, IPSS‐M includes somatic mutations in 31 genes to provide a risk score. In addition, in previous studies, certain genes (*TP53*, *EZH2*, *ETV6*, *RUNX1*, *ASXL1*, and *BOCR*) were associated with an unfavorable prognosis in MDS [26, 27]. On the basis of the above research, Bernard E et al. [7] further divided *SF3B1* ^*mut*^ MDS into three independent groups: *SF3B1* ^*5q*^, *SF3B1* ^*α*^, and *SF3B1* ^*β*^, and found the favorable outcomes were only confined to the *SF3B1* ^*α*^ group without *BCOR* mutation, *BCORL1* mutation, *RUNX1* mutation, *NRAS* mutation, *STAG2* mutation, *SRSF2* mutation, and del (5q). Our study revealed that only 17 patients (15.9%) carried *SF3B1* as the only mutation in our study. The *TET2*, *ASXL1*, *TP53*, and *DNMT3A* genes were the most frequently co‐occurring mutations with the *SF3B1* mutation in our cohort. Therefore, a comprehensive analysis of molecular genetics is important to get a better understanding of the influence of concurrent mutations on prognosis in MDS with the *SF3B1* mutation.

In our study, we analyzed the differences in the number of mutations and the most frequent co‐occurring mutated genes in *SF3B1* ^*mut*^ patients with distinct hotspots. We observed that *SF3B1* ^*mut*^ patients with the *E622* mutation were connected to a small number of mutant genes, further supporting the favorable prognosis of the *E622* hotspot. It is common knowledge that double‐splicing gene mutations are mutually exclusive. In line with the above, we only observed three patients with double‐splicing gene mutations. According to the previous study, *TET2*, *DNMT3A*, and *ASXL1* mutations showed no significant effect on the survival of *SF3B1* ^*mut*^ MDS patients [7]. In line with the above results, Song et al. [28] found that the co‐occurrence of *SF3B1* with *TET2* had a similar prognosis compared to the single *SF3B1* mutation, suggesting the effect of the *TET2* mutation on the prognosis of *SF3B1* ^*mu*t^ MDS was minimal. Song et al. [29] found that the positive prognosis of the *SF3B1* mutation was not affected by the presence of the *DNMT3A* mutation. In addition to the above results, some researchers have demonstrated the negative prognosis of the co‐occurrence of *SF3B1* with *ASXL1* [30]. In our study, patients with *SF3B1/TET2, SF3B1/DNMT3A*, and *SF3B1/ASXL1* co‐mutations showed limited effects on patients with the *SF3B1* mutation. *TP53* mutations are associated with complex karyotypes, transformation to AML, and adverse outcomes [31], which especially applies to MDS with multiple *TP53* hits, since monoallelic patients showed no difference from *TP53* wild‐type patients in outcomes in the analysis of the *TP53* allelic state [32]. However, little is known about the impact of *TP53* concurrent mutations on the prognosis of *SF3B1* ^*mut*^ MDS. In our cohort, we did not find a significantly adverse effect of the *TP53* mutation on clinical features and prognosis in *SF3B1* ^*mut*^ patients. Further analysis showed most of the mutations in *TP53* were mono‐hit in patients with the *SF3B1* mutation. Malcovati et al. [1] proposed the diagnostic criteria for MDS with mutated *SF3B1*, and mutations in *RUNX1* and *EZH2* were the exclusion criteria for the proposed entity. The *RUNX1* mutation was associated with worse OS and a higher AML transformation rate [13, 33], while the co‐occurrence of *SF3B1* with *EZH2* may be associated with more severe anemia and transfusion dependency [34]. In line with the previous studies, the *RUNX1* co‐mutation was an independent adverse prognostic factor in our cohort. However, the *EZH2* co‐mutation showed no discernible effects on OS. Interestingly, we did not observe *RUNX1* and *EZH2* mutations in *SF3B1* ^*mut*^ MDS with the *E622* hotspot, further confirming the favorable prognosis of the *E622* mutation. Previous studies observed no significantly favorable clinical outcome in *SF3B1* ^*mut*^ patients with the *STAG2* mutation [7]. In line with the above results, we found patients with *SF3B1/STAG2* co‐mutations were associated with a shorter OS.

The insufficient cases of concurrent mutations and the heterogeneity of molecular genetics in *SF3B1* ^*mut*^ patients in our study mean that these findings need to be confirmed by analyzing a larger number of cases. Therefore, comprehensive research in molecular genetics is necessary to get a more accurate prognosis for *SF3B1* ^*mut*^ patients with specific subtypes.

The pathogenesis of MDS involves various factors, and it is not yet fully elucidated. With the exception of molecular genetics and cytogenetics, cellular immune disorders have been confirmed to play a role in the pathogenesis of MDS, although the exact mechanism is unknown. Certain mutations in MDS, especially splicing mutations, contribute to immune disorders. According to previous findings, the activation of inflammatory immune signaling has been observed in *SF3B1* ^*mut*^ MDS, leading to the corresponding treatment [35]. T cells play a crucial part in the immune response, and changes in the number and function of T cells may lead to immune dysfunction and the expansion of malignant clones [36, 37]. In patients with MDS, different subtypes and stages may have different T cell polarization states. The T cell immune system is associated with the pathogenesis, maintenance, and progression of MDS. Different T cell subsets perform different functions. CD4^+^ T cells are divided into Th1 and Th2 cells, and the differentiation of Th1/Th2 cells is balanced. Th1 cells mainly mediate cellular immunity and resistance to intracellular pathogens, while Th2 cells mainly mediate humoral immunity and resistance to external pathogens. CD8^+^ T cells are divided into Tc1 and Tc2 cells, which are crucial to the immunologic defense and surveillance of antitumor immunity. NK cells have intrinsic antitumor activity. Previous studies have observed a decrease in NK cell percentage and impaired function of NK cells in patients with MDS, particularly in high‐risk MDS [38]. In addition, the decrease in CD8^+^ T cell percentage is associated with an increased risk of progressing to AML and decreased overall survival [39].

However, the immune characteristics of mutational subtypes in *SF3B1* ^*mut*^ MDS have not been analyzed in other cohorts. To understand more information about the immune features of distinct subtypes, we analyzed the differences in T cell and NK cell percentages in the BM. Interestingly, we found *SF3B1* ^*mut*^ patients with distinct mutation sites showed differences in the polarization of T cells and NK cell percentages. In our study, patients with the *K666* mutation showed significantly lower NK cell percentage and the Th1/Th2 ratio in the BM compared to those with *non‐K666* mutations. Since the polarization of T cells and NK cell percentage are related to antitumor immunity, the decrease in Th1/Th2 ratio and NK cell percentage in the BM in *SF3B1* ^*mut*^ patients with the *K666* mutation may, to some extent, be associated with the poor prognosis in this cohort. These findings indicate that *SF3B1* ^*mut*^ patients with specific mutational status may have unique immune features, which have not been fully confirmed in other cohorts. Based on the activation of immune signaling in the *SF3B1* mutation, we speculate that mutational subtypes in *SF3B1* may cause unique abnormalities in gene splicing, which may result in changes in signaling pathways and proteins, which may influence the production and apoptosis of immune cells by interactions of molecules on the cell surface. A better understanding of how different immune cells and MDS cells interact during disease evolution and how immune cells distribute in different subtypes of MDS is helpful in developing more effective therapy. Therefore, the changes in the function of T cells and NK cells may need to be further explored to improve the prognosis of *SF3B1* ^*mut*^ MDS with unique mutation subtypes.

In summary, *SF3B1* ^*mut*^ patients with specific mutation spots and concomitant gene mutations showed distinct clinical features and prognosis. Therefore, a comprehensive study of molecular genetics, cytogenetics, and immunity contributes to a better understanding of the pathogenesis, risk stratification, and individualized therapy, which is of great significance to improving the prognosis of patients with *SF3B1* mutations.

## Author Contributions

**Qi Liu:** data curation (equal), formal analysis (lead), writing – original draft (lead). **Fanhuan Xu:** data curation (equal). **Juan Guo:** data curation (equal). **Feng Xu:** data curation (equal). **Xinhui Huang:** data curation (equal). **Jianan Chen:** data curation (equal). **Jiacheng Jin:** data curation (equal). **Liyu Zhou:** data curation (equal). **Qi He:** data curation (equal). **Dong Wu:** data curation (equal). **Luxi Song:** data curation (equal). **Zheng Zhang:** data curation (equal). **Cha Guo:** data curation (equal). **Jiying Su:** data curation (equal). **Yumei Zhang:** data curation (equal). **Meng Yan:** data curation (equal). **Chunkang Chang:** data curation (equal). **Xiao Li:** data curation (equal). **Lingyun Wu:** conceptualization (lead), data curation (lead), funding acquisition (lead), project administration (lead), writing – review and editing (lead).

## Ethics Statement

The study was approved by the Ethics Committee of Shanghai Sixth People's Hospital Affiliated to Shanghai Jiao Tong University School of Medicine. All procedures performed in studies involving human participants were in accordance with the ethical standards of the institutional ethics board of Shanghai Sixth People's Hospital Affiliated to Shanghai Jiao Tong University School of Medicine and were in accordance with the 1964 Helsinki Declaration and its later amendments or comparable ethical standards.

## Consent

Informed consent was obtained from all individual participants included in the study.

## Conflicts of Interest

The authors declare no conflicts of interest.

## Supporting information


Data S1.

## Data Availability

The datasets generated and analyzed during the current study are available from the corresponding author on reasonable request.
