# Correction to “Significance of Mutation Spots and Concurrent Gene Mutations on Prognosis and Clinical Outcomes in Myelodysplastic Syndromes with SF3B1 Mutation”

**DOI:** 10.1002/cam4.71139

**Published:** 2025-08-04

Liu Q, Xu F, Guo J, et al. Significance of mutation spots and concurrent gene mutations on prognosis and clinical outcomes in myelodysplastic syndromes with SF3B1 mutation. *Cancer Medicine*, 2025; 14:e70930.

On the seventh line of the “Targeted Gene Sequencing” section, the word “cDNA” was incorrect. This should have read: “gDNA.”

We apologize for this error.

