# Supplementary material for: Significance of Mutation Spots and Concurrent Gene Mutations on Prognosis and Clinical Outcomes in Myelodysplastic Syndromes With SF3B1 Mutation
Source: Cancer Med. 2025 May 8;14(9):e70930. doi: 10.1002/cam4.70930 (PMC12060130; doi:10.1002/cam4.70930)
Supplement: Supplementary file 1 — Data S1. [file CAM4-14-e70930-s001.docx]

**Significance of mutation spots and concurrent gene mutations on prognosis and clinical outcomes in myelodysplastic syndromes with *SF3B1* mutation**

**Journal: Cancer Medicine**

**Authors:**

Qi LiuP^1^, Fanhuan Xu^1^, Juan GuoP^1^, Feng XuP^1^, Xinhui Huang^1^, Jianan Chen^1^, Jiacheng Jin^1^, Liyu ZhouP^1,2^, Qi HeP^1^P, Dong WuP^1^, Luxi SongP^1^,P Zheng ZhangP^1^P, Cha Guo^1^, Jiying SuP^1^P, Yumei Zhang P^2^, Meng Yan P^2^, Chunkang ChangP^1*^P, Xiao Li P^1*^, Lingyun WuP^1, 2*^

**Author affiliation:**

P^1^PDepartment of Hematology, Shanghai Sixth People’s Hospital Affiliated to Shanghai Jiao Tong University School of Medicine, Shanghai, 200233, China.

P^2^PDepartment of Hematology, Shanghai Eighth People’s Hospital, Shanghai, 200233, China.

^*^**Corresponding author:**P

Lingyun Wu, MD, PhD, Department of Hematology, Shanghai Sixth People’s Hospital Affiliated to Shanghai Jiao Tong University School of Medicine, Shanghai, 200233, China.

1. mail: lincy2032@sjtu.edu.cn, Tel: +86-021-24058336, Fax: +86-021-64701361; Xiao Li, MD, PhD, lixiao3326@126.com; Chunkang Chang, MD, PhD,  Changchunkang@yeah.net .

**Supplementary Fig. 1** The difference in the VAF in *SF3B1^mut^* MDS patients with distinct mutation spots


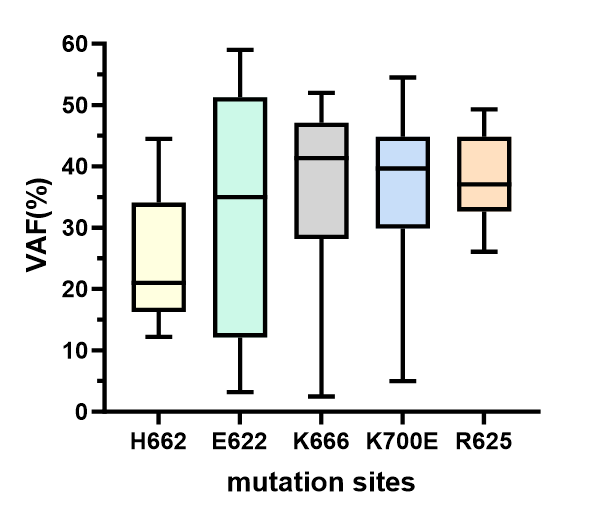


**Supplementary Fig. 2** Concomitant gene mutations of mutation spots of *SF3B1^mut^* MDS


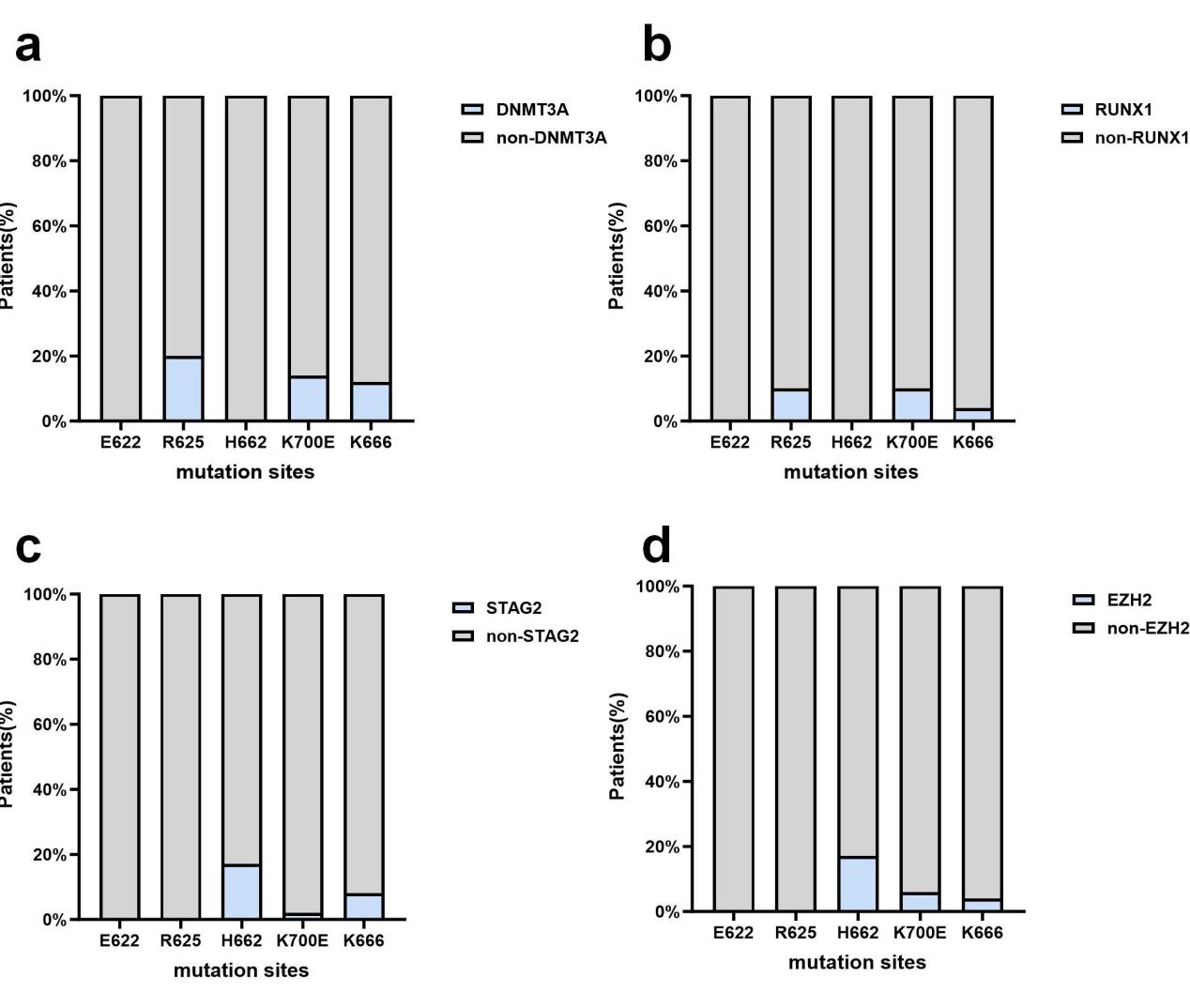


1. The proportion of *DNMT3A* mutation in *SF3B1^mut^* MDS patients with distinct mutation spots. **(b)** The proportion of *RUNX1* mutation in *SF3B1^mut^* MDS patients with distinct mutation spots. **(c)** The proportion of *STAG2* mutation in *SF3B1^mut^* MDS patients with distinct mutation spots. **(d)** The proportion of *EZH2* mutation in *SF3B1^mut^* MDS patients with distinct mutation spots

**Supplementary Fig. 3** Clinical characteristics of concomitant gene mutations in *SF3B1^mut^* MDS


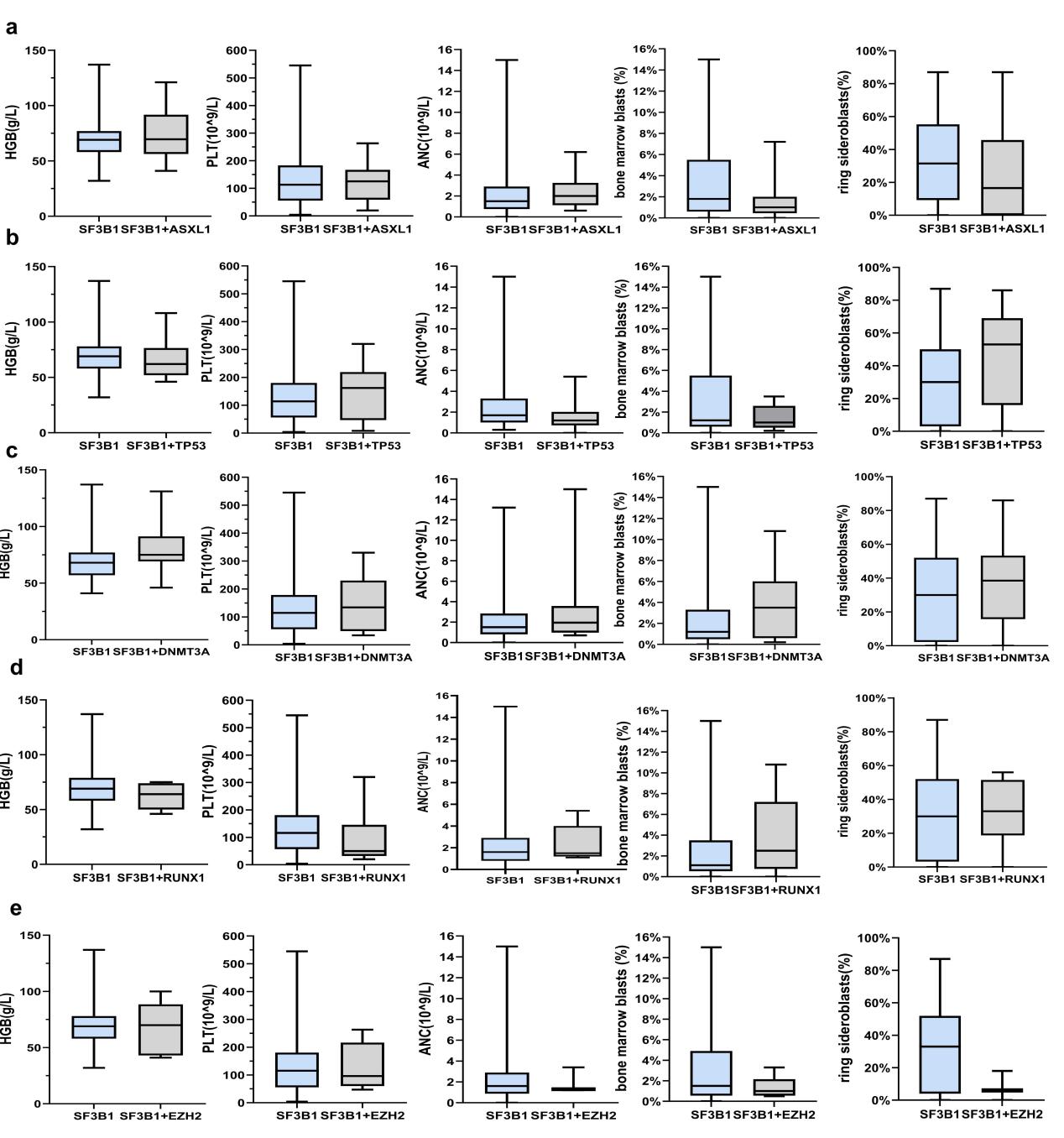


1. The clinical characteristics of *SF3B1^mut^* MDS patients with the *ASXL1* mutation. **(b)** The clinical characteristics of *SF3B1^mut^* MDS patients with the *TP53* mutation. **(c)** The clinical characteristics of *SF3B1^mut^* MDS patients with the *DNMT3A* mutation. **(d)** The clinical characteristics of *SF3B1^mut^* MDS patients with the *RUNX1* mutation. **(e)** The clinical characteristics of *SF3B1^mut^* MDS patients with the *EZH2* mutation.

**Supplementary Fig. 4** The VAF in *SF3B1^mut^* MDS with concomitant gene mutations.


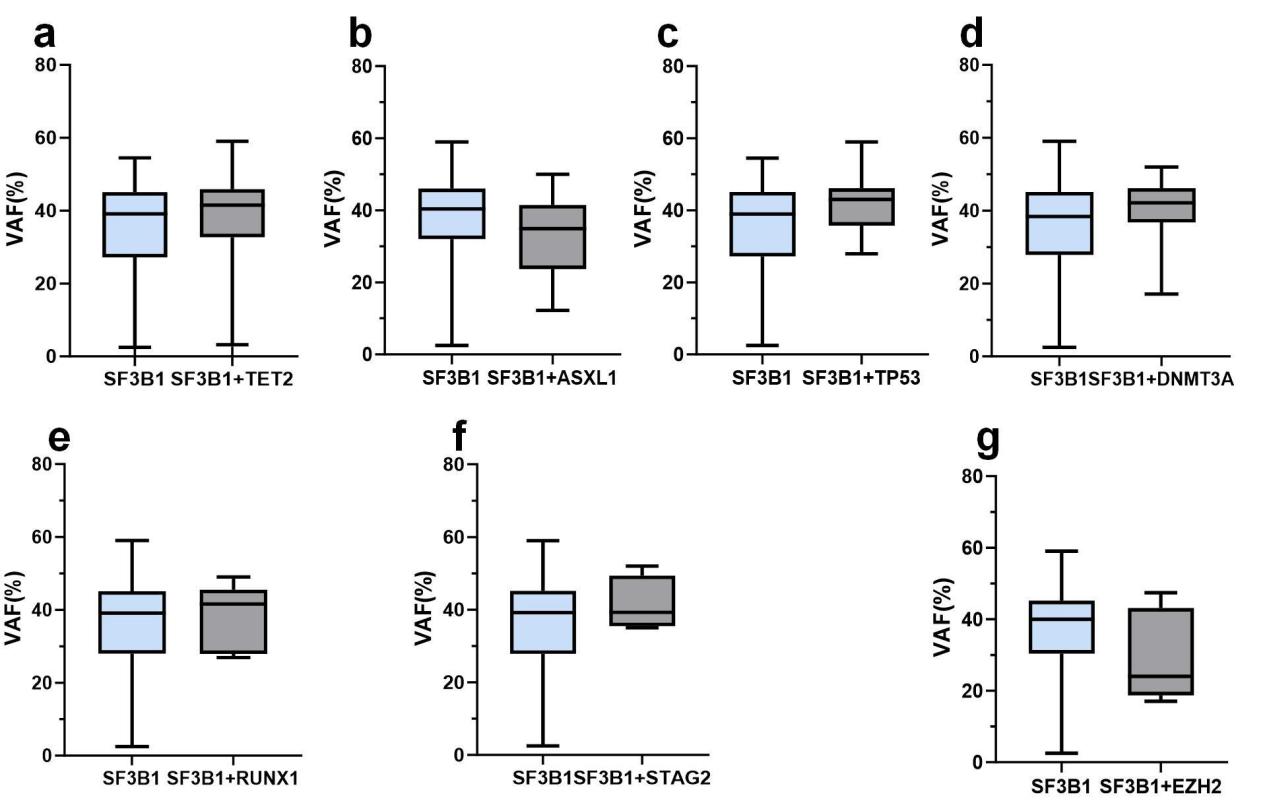


**(a)** The VAF in *SF3B1^mut^* MDS patients with the *TET2* mutation. **(b)** The VAF in *SF3B1^mut^* MDS patients with the *ASXL1* mutation. **(c)** The VAF in *SF3B1^mut^* MDS patients with the *TP53* mutation. **(d)** The VAF in *SF3B1^mut^* MDS patients with the *DNMT3A* mutation. **(e)** The VAF in *SF3B1^mut^* MDS patients with the *RUNX1* mutation. **(f)** The VAF in *SF3B1^mut^* MDS patients with the *STAG2* mutation. **(g)** The VAF in *SF3B1^mut^* MDS patients with the *EZH2* mutation.

**Supplementary Table 1** Clinical characteristics of mutation sites in *SF3B1^mut^* MDS

| Characteristic | *E622*, n=5 | *R625*, n=10 | *H662*, n=6 | *K700E*, n=51 | *K666*, n=26 | *P* |
| --- | --- | --- | --- | --- | --- | --- |
| **Serum ferritin** |  |  |  |  |  | 0.245 |
| Median (ng/mL) | 837.9 | 941.2 | 870.6 | 889.0 | 497.1 |  |
| Range (ng/mL) | 801.9-- | 477.0-1423.8 | 653.2-2066.3 | 438.5-1715.0 | 182.5-1058.5 |  |
| **Serum erythropoietin** |  |  |  |  |  | 0.591 |
| Median (mIU/ml) | 136.5 | 787.0 | 467.5 | 941.0 | 985.5 |  |
| Range (mIU/ml) | 135.0-- | 188.0-2229.0 | 137.4-3214.0 | 293.5-2253.5 | 67.4-2358.0 |  |
| **Cytogenetics** |  |  |  |  |  | 0.717 |
| Normal, n (%) | 3 (60.0) | 3 (30.0) | 3 (50.0) | 21 (41.2) | 13 (50.0) |  |
| del(5q), n (%) | 0 (0.0) | 1 (10.0) | 1 (16.7) | 3 (5.9) | 0 (0.0) |  |
| del(20q), n (%) | 0 (0.0) | 0 (0.0) | 0 (0.0) | 4 (7.8) | 2 (7.7) |  |
| +8, n (%) | 0 (0.0) | 0 (0.0) | 1 (16.7) | 3 (5.9) | 2 (7.7) |  |
| -7, n (%) | 0 (0.0) | 0 (0.0) | 0 (0.0) | 0 (0.0) | 1 (3.8) |  |
| t(3q)/inv(3)/del(3q), n(%) | 0 (0.0) | 0 (0.0) | 0 (0.0) | 2 (3.9) | 0 (0.0) |  |
| Complex karyotype,  n (%) | 0 (0.0) | 1 (10.0) | 1 (16.7) | 4 (7.8) | 1 (3.8) |  |
| others, n (%) | 2 (40.0) | 3 (30.0) | 0 (0.0) | 12 (23.5) | 3 (11.5) |  |
| NA, n (%) | 0 (0.0) | 2 (20.0) | 0 (0.0) | 2 (3.9) | 4 (15.4) |  |
| **WHO 2016 subtype** |  |  |  |  |  | 0.576 |
| MDS-SLD, n (%) | 1 (20.0) | 1 (11.1) | 0 (0.0) | 0 (0.0) | 2 (8.3) |  |
| MDS-MLD, n (%) | 0 (0.0) | 1 (11.1) | 2 (33.3) | 4 (8.0) | 3 (12.5) |  |
| MDS-RS ,n (%) | 3 (60.0) | 4 (44.4) | 2 (33.4) | 35 (70.0) | 12 (50.0) |  |
| MDS-5q, n (%) | 0 (0.0) | 1 (11.1) | 0 (0.0) | 0 (0.0) | 0 (0.0) |  |
| MDS-EB1, n (%) | 0 (0.0) | 2 (22.2) | 2 (33.3) | 7 (14.0) | 2 (8.3) |  |
| MDS-EB2, n (%) | 1 (20.0) | 0 (0.0) | 0 (0.0) | 4 (8.0) | 4 (16.7) |  |
| MDS-U | 0 (0.0) | 0 (0.0) | 0 (0.0) | 0 (0.0) | 1 (4.2) |  |
| **IPSS-R** |  |  |  |  |  | 0.191 |
| Very low, n (%) | 0 (0.0) | 1 (14.3) | 1 (20.0) | 1 (2.1) | 1 (5.3) |  |
| Low, n (%) | 1 (20.0) | 2 (28.6) | 3 (60.0) | 18 (38.3) | 4 (21.1) |  |
| Intermediate, n (%) | 4 (80.0) | 2 (28.6) | 0 (0.0) | 18 (38.3) | 7 (36.8) |  |
| High, n (%) | 0 (0.0) | 0 (0.0) | 0 (0.0) | 4 (8.5) | 5 (26.3) |  |
| Very high, n (%) | 0 (0.0) | 2 (28.6) | 1 (20.0) | 6 (12.8) | 2 (10.5) |  |

Abbreviations: del(5q), deletions of the long arm of chromosome 5; del(20q), deletions of the long arm of chromosome 20; +8, trisomy 8; -7, loss of chromosome 7; t(3q), translocation 3q; inv(3), inversion 3q; del(3q), deletion 3q; NA, not available; WHO, World Health Organization; MDS-SLD, MDS with single lineage dysplasia; MDS-MLD, MDS with multilineage dysplasia; MDS-RS, MDS with ring sideroblasts; MDS-5q, MDS with isolated del (5q); MDS-EB1, MDS with excess blasts-1; MDS-EB2, MDS with excess blasts-2; MDS-U, MDS unclassifiable; IPSS-R, revised international prognostic scoring system

**Supplementary Table 2**  Clinical characteristics of mutation sites in *SF3B1^mut^* MDS with normal karyotype

| Characteristic | *E622*, n=3 | *R625*, n=3 | *H662*, n=3 | *K700E*, n=21 | *K666*, n=13 | *P* |
| --- | --- | --- | --- | --- | --- | --- |
| **HGB** |  |  |  |  |  | 0.572 |
| Median (g/L) | 69.0 | 62.0 | 76.0 | 70.5 | 69.0 |  |
| Range (g/L) | 59.0-- | 58.0-- | 76.0-- | 63.3-78.5 | 57.0-99.0 |  |
| **PLT** |  |  |  |  |  | 0.135 |
| Median (10^9/L) | 160.0 | 249.5 | 260.0 | 148.0 | 86.0 |  |
| Range (10^9/L) | 4.0-- | 180.0-- | 116.0-- | 71.3-191.5 | 27.5-151.5 |  |
| **ANC** |  |  |  |  |  | 0.485 |
| Median (10^9/L) | 0.80 | 3.85 | 2.80 | 1.90 | 1.60 |  |
| Range (10^9/L) | 0.60-- | 1.50-- | 2.20-- | 1.35-3.10 | 0.85-4.80 |  |
| **BM blasts** |  |  |  |  |  | 0.202 |
| Median (%) | 0.80 | 0.80 | 0.50 | 1.00 | 2.75 |  |
| Range (%) | 0.20-- | 0.60-- | 0.40-- | 0.45-2.50 | 1.25-9.95 |  |
| **Number of mutant genes** |  |  |  |  |  | 0.168 |
| 1, n (%) | 1 (33.3) | 0 (0.0) | 1 (33.3) | 3 (14.3) | 1 (7.7) |  |
| 2, n (%) | 2 (66.7) | 2 (66.7) | 1 (33.3) | 9 (42.9) | 2 (15.4) |  |
| ≥ 3, n (%) | 0 (0.0) | 1 (33.3) | 1 (33.3) | 9 (42.9) | 10 (76.9) |  |
| **VAF** |  |  |  |  |  | 0.455 |
| Median (%) | 35.00 | 38.00 | 32.4 | 42.00 | 45.30 |  |
| Range (%) | 3.20-- | 34.30-- | 20.30-- | 29.45-46.75 | 39.00-47.55 |  |
| **TET2** |  |  |  |  |  | **0.025** |
| TET2, n (%) | 1 (33.3) | 3 (100.0) | 0 (0.0) | 3 (14.3) | 2 (15.4) |  |
| Non-TET2, n (%) | 2 (66.7) | 0 (0.0) | 3 (100.0) | 18 (85.7) | 11 (84.6) |  |
| **ASXL1** |  |  |  |  |  | 0.767 |
| ASXL1, n (%) | 0 (0.0) | 0 (0.0) | 1 (33.3) | 3 (14.3) | 3 (23.1) |  |
| Non-ASXL1, n (%) | 3(100.0) | 3 (100.0) | 2 (66.7) | 18 (85.7) | 10 (76.9) |  |
| **TP53** |  |  |  |  |  | 0.925 |
| TP53, n (%) | 0 (0.0) | 0 (0.0) | 0 (0.0) | 2 (9.5) | 1 (7.7) |  |
| Non-TP53, n (%) | 3 (100.0) | 3 (100.0) | 3 (100.0) | 19 (90.5) | 12 (92.3) |  |
| **DNMT3A** |  |  |  |  |  | 0.867 |
| DNMT3A, n (%) | 0 (0.0) | 0 (0.0) | 0 (0.0) | 3 (14.3) | 1 (7.7) |  |
| Non- DNMT3A, n (%) | 3 (100.0) | 3 (100.0) | 3 (100.0) | 18 (85.7) | 12 (92.3) |  |
| **RUNX1** |  |  |  |  |  | 0.867 |
| RUNX1, n (%) | 0 (0.0) | 0 (0.0) | 0 (0.0) | 3 (14.3) | 1 (7.7) |  |
| Non- RUNX1, n (%) | 3 (100.0) | 3 (100.0) | 3 (100.0) | 18 (85.7) | 12 (92.3) |  |
| **STAG2** |  |  |  |  |  | 0.169 |
| STAG2, n (%) | 0 (0.0) | 1 (33.3) | 0 (0.0) | 0 (0.0) | 1 (7.7) |  |
| Non- STAG2, n (%) | 3 (100.0) | 2 (66.7) | 3 (100.0) | 21 (100.0) | 12 (92.3) |  |
| **EZH2** |  |  |  |  |  | 0.379 |
| EZH2, n (%) | 0 (0.0) | 0 (0.0) | 1 (33.3) | 1 (4.8) | 0 (0.0) |  |
| Non- EZH2, n (%) | 3 (100.0) | 3 (100.0) | 2 (66.7) | 20 (95.2) | 13 (100.0) |  |
| **WHO 2016 subtype** |  |  |  |  |  | 0.487 |
| MDS-SLD, n (%) | 0 (0.0) | 0 (0.0) | 0 (0.0) | 0 (0.0) | 1 (8.3) |  |
| MDS-MLD, n (%) | 0 (0.0) | 0 (0.0) | 0 (0.0) | 0 (0.0) | 1 (8.3) |  |
| MDS-RS n (%) | 2(66.7) | 2 (100.0) | 2(66.7) | 18(85.7) | 5 (41.7) |  |
| MDS-5q, n (%) | 0 (0.0) | 0 (0.0) | 0 (0.0) | 0 (0.0) | 0 (0.0) |  |
| MDS-EB1, n (%) | 0 (0.0) | 0 (0.0) | 1 (33.3) | 2 (9.5) | 2 (16.7) |  |
| MDS-EB2, n (%) | 1 (33.3) | 0 (0.0) | 0 (0.0) | 1 (4.8) | 3 (25.0) |  |
| MDS-U | 0 (0.0) | 0 (0.0) | 0 (0.0) | 0 (0.0) | 0 (0.0) |  |
| **IPSS-R** |  |  |  |  |  | 0.108 |
| Very low, n (%) | 0 (0.0) | 0 (0.0) | 1 (33.3) | 0 (0.0) | 1 (8.3) |  |
| Low, n (%) | 1 (33.3) | 2 (100.0) | 1 (33.3) | 14 (70.0) | 3 (25.0) |  |
| Intermediate, n (%) | 2 (66.7) | 0 (0.0) | 0 (0.0) | 5 (25.0) | 4 (33.3) |  |
| High, n (%) | 0 (0.0) | 0 (0.0) | 0 (0.0) | 1 (5.0) | 3 (25.0) |  |
| Very high, n (%) | 0 (0.0) | 0 (0.0) | 0 (0.0) | 0 (0.0) | 1 (8.3) |  |
| **Status** |  |  |  |  |  | 0.172 |
| AML transformation,  n (%) | 0 (0.0) | 0 (0.0) | 1 (33.3) | 1 (5.0) | 4 (30.8) |  |
| Non-AML transformation, n (%) | 3 (100.0) | 2 (100.0) | 2 (66.7) | 19 (95.0) | 9 (69.2) |  |

Abbreviations: HGB, hemoglobin; ANC, absolute neutrophil count; PLT, platelet; RS, ringed sideroblasts; BM, bone marrow; VAF, variant allele frequency; del(5q), deletions of the long arm of chromosome 5; del(20q), deletions of the long arm of chromosome 20; +8, trisomy 8; -7, loss of chromosome 7; t(3q), translocation 3q; inv(3), inversion 3q; del(3q), deletion 3q; NA, not available; WHO, World Health Organization; MDS-SLD, MDS with single lineage dysplasia; MDS-MLD, MDS with multilineage dysplasia; MDS-RS, MDS with ring sideroblasts; MDS-5q, MDS with isolated del (5q); MDS-EB1, MDS with excess blasts-1; MDS-EB2, MDS with excess blasts-2; MDS-U, MDS unclassifiable; IPSS-R, revised international prognostic scoring system; AML, acute myeloid leukemia
